# Changes in the plasma proteome at asymptomatic and symptomatic stages of autosomal dominant Alzheimer’s disease

**DOI:** 10.1038/srep29078

**Published:** 2016-07-06

**Authors:** Julia Muenchhoff, Anne Poljak, Anbupalam Thalamuthu, Veer B. Gupta, Pratishtha Chatterjee, Mark Raftery, Colin L. Masters, John C. Morris, Randall J. Bateman, Anne M. Fagan, Ralph N. Martins, Perminder S. Sachdev

**Affiliations:** 1 Centre for Healthy Brain Ageing, School of Psychiatry, University of New South Wales, Sydney, New South Wales, Australia; 2 Bioanalytical Mass Spectrometry Facility, University of New South Wales, Sydney, New South Wales, Australia; 3 School of Medical Sciences, University of New South Wales, Sydney, New South Wales, Australia; 4 Centre of Excellence for Alzheimer’s disease Research & Care, School of Medical Sciences, Edith Cowan University, Joondalup, Western Australia, Australia; 5 Sir James McCusker Alzheimer’s Disease Research Unit (Hollywood Private Hospital), Perth, Western Australia, Australia; 6 School of Psychiatry and Clinical Neurosciences, University of Western Australia, Perth, Western Australia, Australia; 7 University of Melbourne, Melbourne, Victoria, Australia; 8 Department of Neurology, Washington University School of Medicine, St. Louis, USA; 9 Knight Alzheimer’s Disease Research Center at Washington University School of Medicine, St. Louis, USA; 10 Department of Pathology and Immunology, Washington University School of Medicine, St. Louis, USA; 11 Neuropsychiatric Institute, Prince of Wales Hospital, Sydney, New South Wales, Australia

## Abstract

The autosomal dominant form of Alzheimer’s disease (ADAD) is far less prevalent than late onset Alzheimer’s disease (LOAD), but enables well-informed prospective studies, since symptom onset is near certain and age of onset is predictable. Our aim was to discover plasma proteins associated with early AD pathology by investigating plasma protein changes at the asymptomatic and symptomatic stages of ADAD. Eighty-one proteins were compared across asymptomatic mutation carriers (aMC, *n* = 15), symptomatic mutation carriers (sMC, *n* = 8) and related noncarriers (NC, *n* = 12). Proteins were also tested for associations with cognitive measures, brain amyloid deposition and glucose metabolism. Fewer changes were observed at the asymptomatic than symptomatic stage with seven and 16 proteins altered significantly in aMC and sMC, respectively. This included complement components C3, C5, C6, apolipoproteins A-I, A-IV, C-I and M, histidine-rich glycoprotein, heparin cofactor II and attractin, which are involved in inflammation, lipid metabolism and vascular health. Proteins involved in lipid metabolism differed only at the symptomatic stage, whereas changes in inflammation and vascular health were evident at asymptomatic and symptomatic stages. Due to increasing evidence supporting the usefulness of ADAD as a model for LOAD, these proteins warrant further investigation into their potential association with early stages of LOAD.

Due to the ageing of the population in most countries, dementia is a rapidly growing problem with cases predicted to rise to about 115 million worldwide by 2050^1^. The most common cause of dementia is Alzheimer’s disease (AD), for which there is at present no cure. It is hypothesised that several decades-long asymptomatic pathological processes lead up to the clinical manifestation of AD^2^, however, these early pathological processes are still poorly defined.

Identification of the processes and associated biochemical changes leading up to symptomatic AD is a difficult task as it cannot be predicted who will develop late onset AD (LOAD) in the future. By contrast, the autosomal dominant form of AD (ADAD) has near absolute certainty of onset in mutation carriers (MC) and the age at onset (AAO) is also predictable based on family history^3^. This enables well-informed prospective studies to identify early changes associated with AD pathology. Furthermore, the relatively young age of ADAD patients minimises age-related changes and co-morbidities, which can confound studies in older adults.

ADAD is caused by mutations that effect alterations in amyloid precursor protein (APP) processing. Mutations have been identified in three genes [*APP*, NCBI Entrez Gene 351, presenilin 1 (*PSEN1*, NCBI Entrez Gene 5663), or presenilin 2 (*PSEN2*, NCBI Entrez Gene 5664)] and provide a defined change in biochemical pathways that can be studied. Less than 1% of all AD cases are attributed to ADAD, but increasing evidence suggests that the clinical and neuropathological features of ADAD and LOAD are similar, encouraging the use of ADAD as a model for AD in general^2^,^4^,^5^.

Cerebrospinal fluid (CSF) has been the major focus for tracking biochemical changes related to AD. Several studies measured CSF and plasma amyloid-β (Aβ) and tau species as well as CSF proteins in ADAD MC^2^,^4^,^10^,^11^,^12^,^13^, however, to the best of our knowledge, discovery-based proteomics have not yet been applied to blood samples from ADAD MC. Blood is readily accessible and routinely used in tracking onset and progression of a variety of diseases involving peripheral organs. Approximately 500 mL CSF are absorbed into the blood daily, making it a credible resource for studying brain disorders. It is also likely that the blood-brain barrier is compromised early on in AD^6^, potentially allowing brain-associated molecules to enter the blood stream. There is mounting evidence to indicate that protein (and lipid) changes in the periphery reflect the asymptomatic, prodromal and symptomatic stages of LOAD^7^,^8^,^9^, but how this compares to plasma protein changes in the asymptomatic and symptomatic stages of ADAD is currently unknown.

In this pilot study on a small number of samples, we used the unbiased comparative proteomics technique of isobaric tags for relative and absolute quantitation (iTRAQ) to compare the plasma protein profiles of ADAD MC at asymptomatic and symptomatic stages with related noncarriers (NC) with the aim to explore disease mechanisms and discover proteins associated with early stages of AD pathology. Each of the 81 proteins quantified was tested for associations with cognitive measures, brain amyloid deposition, glucose metabolism and brain volumetrics. Proteins differentially abundant in MC and NC and associated with the measures above indicate the pathways that are affected by AD pathology at the asymptomatic and symptomatic stages.

## Results

### Cohort characteristics

Participant characteristics are presented in Table 1. The mutation types of the 35 participants included various *PSEN1* mutations (ΔE9, Q222H, H163R, I436V, L271V, M233T, S169L, S170F and S290C) from 11 pedigrees and the *APP*E693Q (Dutch mutation) from one pedigree. The participants were grouped according to cognitive and mutation status into NC (*n* = 12), aMC (CDR score of 0, *n* = 15) and sMC (CDR score ≥0.5, *n* = 8). One participant in the NC group had a CDR of 0.5 due to severe depression. sMC were entirely comprised of *PSEN1* MC. The MMSE ranged from 23 to 30 for NC, 26 to 30 for asymptomatic and 12 to 26 for symptomatic MC. PiB PET data was available for ten NC, 12 aMC and six sMC. PiB PET SUVRs ranged from 0.43 to 0.64 for NC, 0.48 to 2.05 for aMC and 0.45 to 2.72 for sMC. Using a cut off of ≥0.75, none, six and six participants were positive for fibrillar amyloid in the precuneus and/or caudate nucleus in the NC, asymptomatic and symptomatic MC groups, respectively.

### Quantitation of plasma proteins using iTRAQ

iTRAQ enables unbiased quantitative comparison of proteins present in multiple samples by labelling of peptides with isobaric stable isotope tags that fragment upon collision-induced dissociation into reporter ions used for quantitation. A maximum of eight tags are available so that up to eight samples can be multiplexed and analysed at the same time, avoiding run-to-run variation. If more than eight samples are to be analysed, several multiplex experiments are performed, each containing the same reference standard to enable relative quantitation of proteins across all experiments. Here six 8-plex iTRAQ experiments were performed for analysis of the 35 immunodepleted plasma samples. As reference, a pool of plasma samples from all NC was used. Therefore, each iTRAQ multiplex experiment enabled the identification and quantitation of seven protein samples relative to the pooled reference standard.

Supplementary Table S1 summarises the results of the six iTRAQ multiplex experiments, including the number of proteins identified at a local FDR of 1%. Eighty-one proteins were quantified across all 35 plasma samples and used in the statistical analyses. Their relative abundances are given in Supplementary Table S2. The ratio represents the abundance of each protein relative to the same protein in the reference pool that was included in all of the multiplex iTRAQ experiments. The ratio value is used to compare each individual in the NC, aMC and sMC groups (*cf* to using the reference pool itself as baseline for comparisons). Only human proteins with a minimum unused score of 1.3 (≥95% confidence in correct sequence identification) and at least two distinct peptides for protein identification were included.

### Plasma proteins differentially abundant in asymptomatic carriers of *PSEN1* and Dutch mutations and NC

Using a linear model with age, gender, *APOE* ε4 status, EYO, mutation type and status (i.e. NC, *PSEN1* and Dutch mutation groupings) as covariates, we compared the plasma protein profiles of asymptomatic carriers of *PSEN1* and Dutch mutations to investigate potential differences in protein expression in these phenotypically different mutation types. Eight proteins were found to differ significantly between these groups (Supplementary Fig. S1), namely complement C4-A, zinc-α-2-glycoprotein, hemopexin, N-acetylmuramoyl-L-alanine amidase, α-2-antiplasmin, apolipoprotein L1, C1 inhibitor and inter-α-trypsin inhibitor heavy chain H2 (ITIH2). Comparisons of asymptomatic *PSEN1* MC and NC as well as asymptomatic Dutch MC and NC identified 16 and 14 proteins as differentially abundant between these groups, respectively, with three proteins (fibronectin, complement C3 and α-2-antiplasmin) common to both comparisons (Supplementary Table S3). The effect size ranged from −1.23 for apolipoprotein L1 to 1.64 for complement C4-A. However, the sample numbers of *PSEN1* and Dutch aMC are very small (*PSEN1 n* = 6, Dutch *n* = 9) and all Dutch MC are from the same family. Hence, this has to be considered an exploratory analysis that requires replication in a larger sample.

### Plasma proteins differentially abundant in NC, aMC and sMC

Using a linear model with age, gender, *APOE* ε4 status, EYO, mutation and diagnostic status (i.e. NC, aMC and sMC groupings) as covariates, we compared the plasma protein profiles of NC, aMC and sMC. Eighteen proteins differed significantly (*q* < 0.05) in the global test of abundance in the three groups (Table 2). The effect size ranged from −0.85 for histidine-rich glycoprotein (HRG) to 0.74 for thyroxine-binding globulin (TBG). In total, six of the proteins identified here were also differentially abundant in the above comparison of NC with asymptomatic carriers of *PSEN1* or Dutch mutations (Supplementary Table S3) [lumican, TBG, complement components C3 and C6, heparin cofactor II (HCII) and attractin (ATRN)].

**Figure 1:**
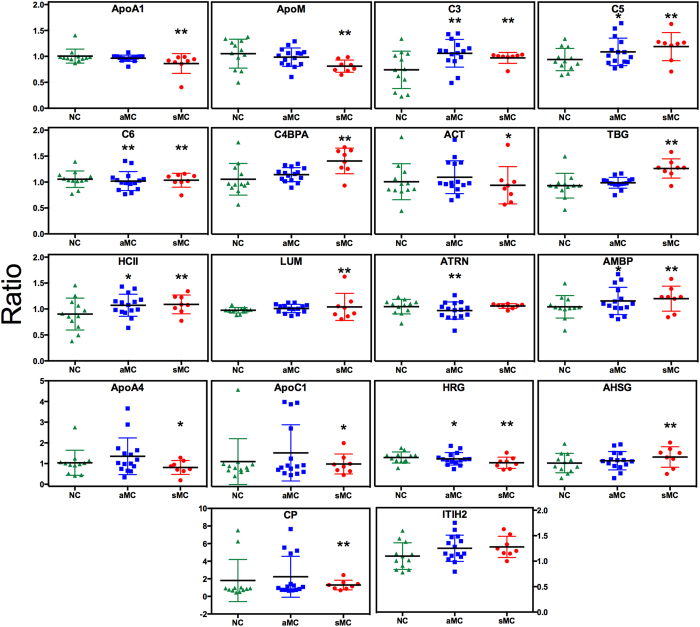
Ratios of differentially abundant proteins. Mean ratios and standard deviations of plasma proteins from noncarriers (NC), asymptomatic (aMC) and symptomatic mutation carriers (sMC) relative to reference masterpool as quantified by iTRAQ. All 18 proteins differed significantly in abundance in a global comparison of NC, aMC and sMC groups (Table 2). Ratios that significantly differed from the NC group in pairwise comparisons are marked with asterisks (*p < 0.05/2 compared to NC; **p < 0.01/2 compared to NC). ApoA1, Apolipoprotein A-I; ApoM, apolipoprotein M; C3, complement component C3; C5, complement component C5; C6, complement component C6; C4BPA, C4-b binding protein α chain; ACT, α-1-antichymotrypsin; TBG, thyroxine-binding globulin; HCII, heparin cofactor II; LUM, lumican; ATRN, attractin; AMBP, protein α-1-microglobulin/bikunin precursor; ApoA4, apolipoprotein A-IV; ApoC1, apolipoprotein C-I; HRG, histidine-rich glycoprotein; AHSG, α-2-HS-glycoprotein; CP, ceruloplasmin; ITIH2, inter-α-trypsin inhibitor heavy chain H2.

Group comparisons were performed to compare the levels of these 18 proteins between NC and aMC as well as NC and sMC. Levels of complement components C3, C5, C6, protein α-1-microglobulin/bikunin precursor, HRG, HCII and ATRN differed significantly (*p* < 0.025) between NC and aMC (Fig. 1). Except for ATRN, the same proteins also differed significantly between NC and sMC, together with the additional proteins lumican, α-1-antichymotrypsin (ACT), TBG, C4b-binding protein α chain, α-2-HS-glycoprotein (AHSG), ceruloplasmin, apolipoproteins A-I (ApoA1), A-IV (ApoA4), C-I (ApoC1) and M (ApoM) (Fig. 1). ITIH2 did not differ significantly in the group comparisons (Fig. 1). The majority of these proteins are involved in inflammation, hemostasis and lipid metabolism (Table 2).

### Plasma proteins associated with cognition and neuroimaging markers

**Figure 2:**
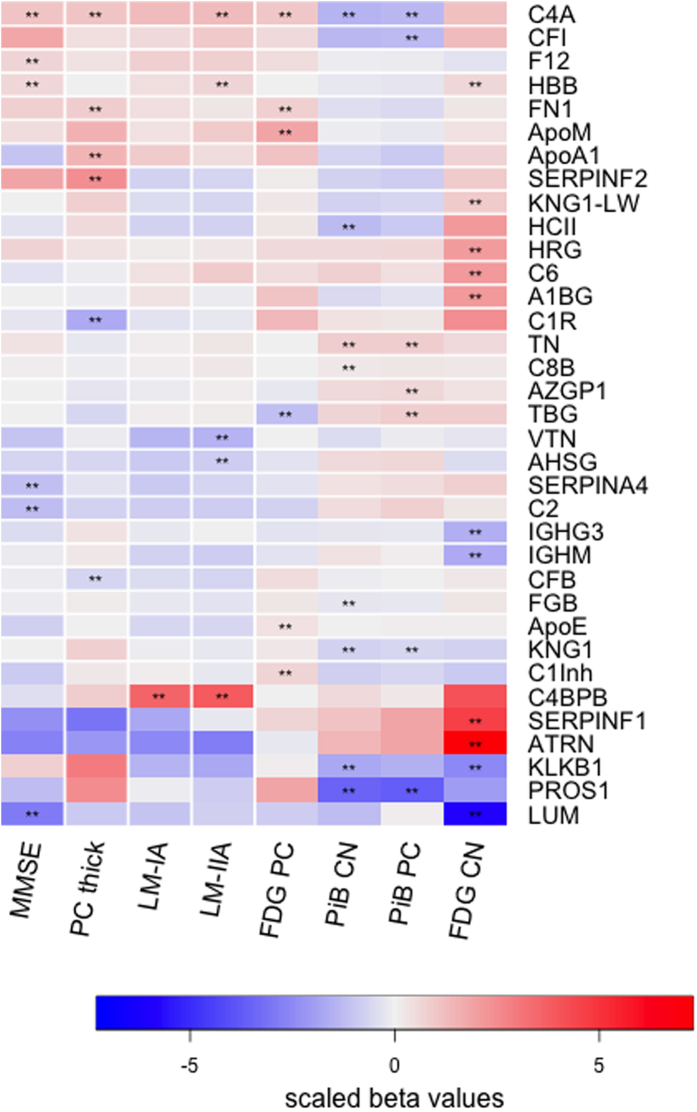
Heat map of proteins associated with cognition and neuroimaging measures. The heat map shows proteins that were significantly associated with at least one of eight outcomes, namely cognitive function represented by MMSE score, episodic memory represented by LM-IA and LM-IIA scores, average precuneus thickness (PC thick), glucose metabolism in the precuneus (FDG PC) and caudate nucleus (FDG CN) and amyloid deposition in the precuneus (PiB PC) and caudate nucleus (PiB CN). Associations marked with ** were found to be significant with a Bonferroni corrected *p* value of < 0.05/81. Colour represents scaled β values with red indicating positive associations and blue negative associations. The actual values for β coefficients, standard errors and *p*-values are given in Table S4. A1BG, α-1B-glycoprotein; AHSG, α-2-HS-glycoprotein; ApoA1, Apolipoprotein A-I; ApoE, apolipoprotein E; ApoM, apolipoprotein M; ATRN, attractin; AZGP1, zinc-α-2-glycoprotein; C1R, complement C1r subcomponent; C2, complement C2; C4A, complement C4-A; C4BPB, C4-b binding protein β chain; C6, complement component C6; C8B, complement component C8; CFB, complement factor B; CFI, complement factor I; TN, tetranectin; F12, coagulation factor XII; FGB, fibrinogen β chain; FN1, fibronectin; HBB, haemoglobin subunit β; HRG, histidine-rich glycoprotein; IGHG3, Ig γ-3 chain C region; IGHM, Ig μ chain C region; KNG1, kininogen; KNG1-LW, low molecular weight isoform of kininogen; LUM, lumican; KLKB1, plasma kallikrein; PROS1, Vitamin K-dependent protein S; SERPINA4, kallistatin; TBG, thyroxine-binding globulin; HCII, heparin cofactor II; SERPINF1, pigment epithelium-derived factor; SERPINF2, α-2-antiplasmin; C1Inh, plasma protease C1 inhibitor; VTN, vitronectin. The heat map was generated using the R package gplots.

The 81 proteins quantified in plasma from all participants were tested for their associations with cognitive function (MMSE score), episodic memory (LM-IA and LM-IIA scores), precuneus thickness (MRI), glucose metabolism (FDG PET) and amyloid deposition (PiB PET) in the precuneus and the caudate nucleus. The precuneus and caudate nucleus were chosen for analyses, as they are known to show early amyloid deposition in ADAD^2^. Thirty-five proteins were significantly associated with at least one of these measures (Fig. 2 and Supplementary Tables S4–S7), including nine proteins that significantly differed in abundance among aMC, sMC and NC groups (Table 2): ApoM and TBG were associated with glucose metabolism in the precuneus, whereas C6, ATRN, HRG and lumican were associated with glucose metabolism in the caudate nucleus. ApoA1 was the only protein of the nine to be associated with precuneus thickness. TBG and HCII were associated with amyloid deposition in the precuneus and caudate nucleus, respectively. Regarding cognitive measures, lumican was associated with MMSE score, and AHSG with episodic memory (LM-IIA score).

The protein with the most significant associations was complement component C4-A (C4A), being linked to two (MMSE and LM-IA) of the three cognitive measures and four (precuneus thickness, glucose metabolism in the precuneus, amyloid deposition in the precuneus and caudate nucleus) of the five neuroimaging measures. C4b-binding protein β chain was strongly associated with both episodic memory scores.

The strongest and most consistent associations for amyloid deposition in the precuneus and caudate nucleus were with C4A, tetranectin, kininogen-1 and Vitamin K-dependent protein S.

### Validation of iTRAQ proteomics using orthogonal method

To verify the quantitative data obtained by iTRAQ proteomics, levels of HCII were quantified by enzyme-linked immunosorbent assay (ELISA) in the low abundance protein fractions derived from all plasma samples and used in the iTRAQ experiments, including the NC reference masterpool. For comparison with the iTRAQ data, results were expressed as the ratio of μg HCII per mg low abundance protein relative to the NC pool (Supplementary Table S8). Quantitation by both methods corresponded well with a correlation coefficient (*r*) of 0.58 (*p* = 0.0003), however, the statistically significant difference in HCII levels between NC and aMC as well as NC and sMC groups was not replicated.

### Protein network analysis

The 18 proteins that were differentially abundant in NC and MC at asymptomatic and symptomatic stages were analysed for molecular functions, biological processes and KEGG pathways that might be enriched against the background of the 81 proteins quantified. However, none of the molecular functions, biological processes or pathways were significantly enriched.

**Figure 3:**
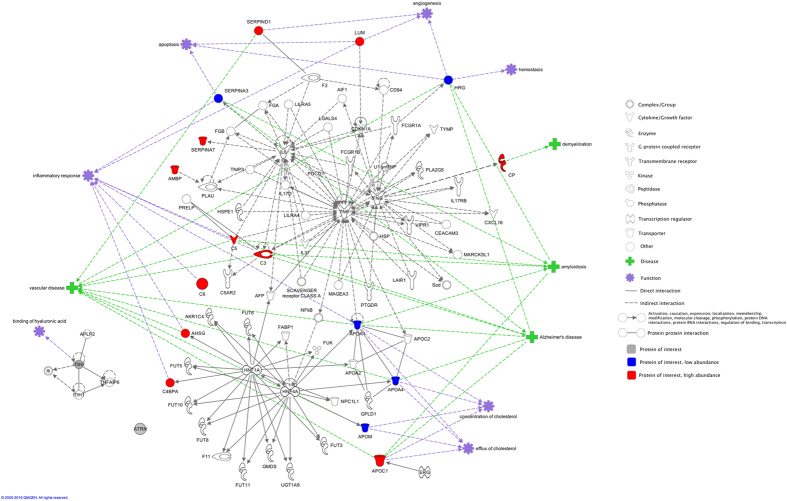
Protein network (IPA) of the proteins differentially abundant in NC, aMC and sMC groups. IPA analysis of the differentially abundant proteins (denoted by their gene names, refer to Table 2), which are coloured in red (increased abundance in sMC vs NC), blue (decreased abundance in sMC vs NC) and grey (differentially abundant in NC, aMC and sMC but not in sMC vs NC comparison). Prevalent functions and disease processes related to the proteins are indicated in the network by purple and green symbols, respectively.

In a network analysis, 14 proteins formed a network associated with AD, vascular disease and inflammatory response (Fig. 3). ApoC1 and C6 did not link to any proteins but instead to the disease processes and functions in the network. ITIH2 and ATRN had no links of any kind to the network, but their respective interactions are also shown in Fig. 3. ITIH2 is connected to a five-protein network related to hyaluronic acid binding.

## Discussion

This preliminary study using a small number of samples identified proteins that were potentially differentially abundant in plasma from asymptomatic and symptomatic carriers of mutations causing ADAD compared to NC. Potential differences in *PSEN1* and Dutch mutation types were also investigated. In addition, proteins were tested for association with cognition and neuroimaging markers in this cohort. To the best of our knowledge this is the first discovery proteomics study using plasma from ADAD MC.

### Plasma protein profiles of asymptomatic *PSEN1* and Dutch mutation carriers

Nine of the aMC in this study carry the Dutch mutation, which is phenotypically distinct from other *PSEN1* or *APP* mutations in that it features more severe cerebral amyloid angiopathy (CAA) and cerebral haemorrhage, but fewer plaques and neurofibrillary tangles^14^,^15^. Therefore, we investigated differences in the plasma protein profiles of asymptomatic carriers of *PSEN1* and Dutch mutations, hypothesising that Dutch MC would show pronounced changes in proteins relating to vascular health. The eight proteins that were differentially abundant between *PSEN1* and Dutch MC are mainly involved in the immune response, inflammation and transport of heme and lipids, but α-2-antiplasmin functions in vascular health as a serine protease inhibitor specific for plasmin, thereby regulating fibrinolysis. Homozygous deficiency in α-2-antiplasmin results in uncontrolled fibrinolysis and subsequent severe haemorrhagic tendencies^16^. Hemopexin and apolipoprotein L1 have tentative links to vascular health, but further studies are needed to confirm these proposed roles. Hemopexin has been suggested to control heme-iron recovery within the brain, where excess heme is associated with intracerebral or subarachnoid hemorrhages and ischemia^17^. Apolipoprotein L1 has been linked to atherosclerosis^18^. Interestingly, α-2-antiplasmin was also differentially abundant in both *PSEN1* aMC to NC as well as Dutch aMC to NC comparisons, whereas hemopexin and apolipoprotein L1 were differentially abundant in *PSEN1* aMC relative to NC, indicating vascular involvement is not exclusive to the Dutch mutation.

Comparisons of asymptomatic *PSEN1* MC to NC as well as Dutch MC to NC showed limited overlap of proteins, which may relate to the phenotypic differences resulting from these two different gene mutations. However, both groups showed similar alterations relative to the NC group in proteins related to vascular health. Four proteins with vascular functions were deregulated in each of the *PSEN1* and Dutch mutation groups and two of these, fibronectin and α-2-antiplasmin, were common to both groups. Kallikrein and kininogen-1, deregulated in *PSEN1* MC relative to NC, as well as kallistatin, deregulated in Dutch MC relative to NC, are all part of the kallikrein-kinin system. Similarly, the biological processes indicated by the other proteins that were differentially abundant in *PSEN1* aMC to NC and Dutch aMC to NC comparisons were alike, suggesting that subtle changes to specific proteins may differentiate these genetic dementia variants, but within the framework of similar biological processes. However, due to the small sample size of both groups and the relatedness of all Dutch MC in this study, this comparison is underpowered from a statistical point of view, and the conclusions will need to be confirmed by future studies, which should include higher sample and pedigree numbers.

Although the above analysis revealed potential subtle differences in the protein profile of *PSEN1* and Dutch aMC, it also showed the common themes of inflammation and vascular changes in both these groups. Both mutation types share the underlying pathology of an altered Aβ metabolism, resulting in CAA and plaques albeit at different rates in the two mutation types. The commonality of the two mutation types might be particularly relevant to the asymptomatic stage of the condition, where the underlying Aβ toxicity has not yet resulted in further pathological manifestations, such as intracerebral haemorrhages in Dutch MC, that might have flow on effects leading to further differentiation in the disease process. Hence, we proceeded to group asymptomatic carriers of *PSEN1* and Dutch MC together for the below analysis of protein changes at the asymptomatic and symptomatic stage of ADAD in order to give a larger sample number and thereby increase robustness.

### Plasma protein profiles at asymptomatic and symptomatic stages of ADAD

As expected, more changes to the plasma protein profile were observed in sMC than in aMC when compared to NC, with only seven proteins differing in abundance between aMC and NC, but 16 between sMC and NC. Evidence suggests that biochemical changes in the periphery begin and are detectable very early in the disease process^2^,^13^. With an average EYO of −8.5 for the aMC group, it is reasonable that we were able to detect some changes in the plasma protein profile of asymptomatic participants, albeit less pronounced than in the sMC group. Except for one protein (ATRN), the same proteins that were deregulated at the asymptomatic stage were also deregulated at the later symptomatic stage, even though the aMC group included Dutch MC whereas the sMC group was exclusively comprised of *PSEN1* MC. These proteins in particular indicate an early and continuous involvement of the immune system. By contrast, several proteins related to lipid metabolism were only differentially abundant in sMC, i.e. at a later stage of the disease. Therefore, alterations in lipid metabolism may be a later event in disease progression relative to inflammation. Interestingly, many of the protein changes we observed also related to vascular health at both asymptomatic (*PSEN1* and Dutch MC) and symptomatic stages (*PSEN1* MC only), including the apolipoproteins, AHSG, HRG, HCII and ACT. Cerebrovascular dysfunction is a common component in LOAD pathology, where it may however be difficult to distinguish from comorbidity. In contrast, there is low comorbidity of vascular disease in the younger ADAD population, strengthening our finding. Changes in the cerebral blood flow of asymptomatic and mildly symptomatic *PSEN1* and *APP* MC also indicate the presence of cerebrovascular dysfunction early in the ADAD disease process^19^.

Although the individual proteins across studies do vary, the overall proteomic changes observed here with regard to protein families were comparable to changes in CSF of ADAD subjects (mostly related to inflammation and synaptic loss)^11^ and plasma from LOAD subjects^20^,^21^ (Supplementary Fig. S2). Sixteen of the 18 differentially abundant proteins identified here have previously been linked to LOAD (see below and^22^,^23^,^24^,^25^ for lumican, ceruloplasmin and TBG). This adds to the increasing evidence from CSF biomarker, amyloid imaging and brain volumetric studies that suggest the usefulness of ADAD as a model for LOAD^2^,^4^,^5^. Two proteins, ATRN and HCII, have not previously been related to AD. ATRN differed significantly in the early stages of disease and showed a strong positive association with glucose metabolism in the caudate nucleus. Its role in immunity, reactive oxygen species (ROS) metabolism and CNS myelination could provide possible links to AD disease mechanisms via inflammation, abnormal ROS metabolism in the brain and spongiform degeneration, which is sometimes associated with AD pathology^26^. HCII differed significantly and consistently in early and later stages of the disease and was inversely associated with amyloid deposition in the caudate nucleus. It has been suggested as a biomarker for arterial disease due to its role in atherosclerosis^27^,^28^,^29^. Hence, it might be indicative of the cerebrovascular component in AD pathology, which is evident in ADAD from an early stage^19^. More details on the individual differentially abundant proteins and their potential role in AD pathology are provided in the Supplementary Discussion, classified by their main functions into inflammation, hemostasis/vascular health and lipid metabolism.

### Proteins associated with cognition and neuroimaging biomarkers

Similar to the proteins whose plasma levels were altered in aMC and sMC, the proteins that associated with any of the cognition or neuroimaging markers mainly have functions in the complement system (complement factors B and I, complement components C2, C4-A, C6, and C8 β chain, complement C1R subcomponent, C4b-binding protein β chain and C1 inhibitor), adaptive immunity (Ig γ-3 chain C region, Ig μ chain C region), hemostasis/vascular health (kininogen-1, α-2-antiplasmin, vitamin K-dependent protein S, C1 inhibitor, fibrinogen β chain, fibronectin, C4b-binding protein β chain, haemoglobin subunit β, ApoA1, HCII, coagulation factor XII and HRG) and lipid metabolism (ApoA1, ApoM, ApoE). ApoE, IgM and TBG, which associated in this study with glucose metabolism in the precuneus, in the caudate nucleus and amyloid deposition in the precuneus, respectively, were also associated with grey matter changes in LOAD brains in a cross-sectional study^25^. In the AIBL cohort, plasma ApoE levels were inversely associated with amyloid deposition measured by PiB PET^30^.

Nine of the proteins identified as differentially abundant in plasma were also associated with cognition or neuroimaging markers (ApoA1, ApoM, HCII, HRG, C6, TBG, AHSG, ATRN, lumican). This overlap between the different outcome measures supports the potential link of these proteins with AD pathology. In particular proteins that associate with brain amyloid deposition, the hallmark feature of AD, might be of interest in future studies. Here the strongest and most consistent associations were with C4A, tetranectin, kininogen-1, vitamin K-dependent protein S, however, none of these proteins were detected at significantly different levels in the plasma of NC, aMC and sMC. This could be due to method limitations, since the iTRAQ methodology is not particularly sensitive to minor changes. Hence these proteins, in particular C4A, which showed the most consistent associations, might still be of interest in future studies and could be investigated with more sensitive quantitative methods (e.g. ELISA). Protein associations with brain glucose metabolism (FDG PET), which is considered to reflect neuronal damage, were less consistent than those with amyloid deposition.

### Study limitations

Limitations to discovery ‘omics’ studies on blood in general are the relatively small number of proteins quantified compared to other approaches or sample types, which is due to the technical challenge posed by the large dynamic range of proteins in blood. Furthermore, proteomics approaches generate data that are quantitative only in a relative not absolute sense and often also insensitive to minor changes. Hence, proteomics in general should be regarded as a discovery tool to generate rather than confirm hypotheses. Thorough follow up verification/validation of the quantitative changes observed is essential and should be carried out in larger populations using targeted, rigorously quantitative approaches such as ELISA multiplex assays and mass spectrometric based multiple reaction monitoring (MRM).

Limitations specific to this study include the small sample size and the relatedness of some individuals, which was controlled for statistically using the GEE method with robust sandwich estimator. However, the robust sandwich estimator is known to perform poorly for small sample sizes, necessitating validation in a larger sample. Furthermore, the quantitative changes observed between NC and aMC or sMC groups (Fig. 1) were statistically significant, yet relatively small. In fact, the statistically significant difference between NC and MC groups detected for HCII in the iTRAQ experiment could not be replicated using an orthogonal method (ELISA) despite the overall adequate correlation of data obtained by the two methods. This could be due to the small sample size, resulting in a large effect of outliers, and/or quantitative differences between the HCII peptides and epitopes detected by iTRAQ and antibody, respectively. This also illustrates that these proteins despite offering valuable insights into pathways associated with ADAD and its progression might not have great potential as biomarkers. As pointed out above for proteomics studies in general, further ELISA or MRM assays and a larger sample would be needed to assess how well the changes we observed in this preliminary study replicate in a larger sample and whether any of the proteins identified here are biomarker candidates. In addition, many of these proteins might lack the specificity required for a biomarker, since for example proteins related to inflammation would be expected to be affected by a wide variety of conditions other than AD.

The strength of our study lies in its truly unbiased discovery approach, providing insights into what processes are involved at certain stages of the disease. Moreover, the young age of this cohort excludes many of the age- and comorbidity-associated confounders common to older LOAD cohorts. The inclusion of as yet asymptomatic participants who are destined to develop AD later in life allows for identification of proteins associated with the very early stages of the disease.

## Conclusion

In summary, the proteomic changes in plasma from carriers of mutations causing ADAD are consistent with the well-established role of inflammation and lipid metabolism in AD pathology. Changes related to inflammation were evident early in asymptomatic individuals; however, changes related to lipid metabolism were only evident at the later symptomatic stage of the disease. Interestingly, the changes also implied a vascular component even at the asymptomatic stage in this young cohort. Cerebrovascular dysfunction is common in LOAD, where it is however difficult to distinguish from a comorbidity in mostly elderly subjects. Because the younger age of the ADAD population and low vascular comorbidity, our findings support a vascular and inflammation process in AD. Due to increasing evidence supporting the usefulness of ADAD as a model for LOAD, we believe the proteins identified here might also be associated with the asymptomatic or prodromal stages of LOAD.

## Materials and Methods

### Study population and blood collection

EDTA plasma samples were obtained from 35 participants of the Dominantly Inherited Alzheimer Network (DIAN) performance sites at the Edith Cowan University, Perth, and the University of Melbourne, Melbourne, Australia. DIAN is an ongoing global, collaborative effort of international AD centres that uses standardised protocols to obtain longitudinal clinical, cognitive, genetic, neuroimaging, biofluid and neuropathological data from asymptomatic and symptomatic MC and their NC family members^31^. Adult children (18 years or older) of parents clinically affected by ADAD are eligible for enrolment in DIAN. In May 2012, the DIAN cohort numbered 255 individuals^31^. Ethics approval was provided by the ethics committees of the Hollywood Private Hospital (WA), Edith Cowan University, the University of Western Australia and the Melbourne Health Human Research Ethics Committee. All experiments and methods were carried out in accordance with the approved guidelines and regulations. All participants, or, in cases of impaired capacity to give consent, their proxies, gave written informed consent before their participation.

Protocols for DIAN blood collection and processing are consistent with the Alzheimer’s Disease Neuroimaging Initiative (ADNI) protocols^32^. Blood was collected into EDTA-coated polypropylene tubes by venepuncture in the morning after overnight fasting. EDTA plasma samples were stored at −80 °C in small volume aliquots to limit the number of freeze/thaw cycles to a maximum of two.

To determine the parental AAO, family members were asked in a semi-structured interview to estimate the age at which the parent’s progressive cognitive decline became evident. The EYO calculated as the age of the participant at time of blood draw minus the parental AAO.

### Sample preparation, iTRAQ-labelling and LC-MSMS

The six most abundant plasma proteins (albumin, transferrin, immunoglobulins G and A, haptoglobin and antitrypsin) were immunodepleted from plasma samples (20 μl) using the Agilent (Santa Clara, USA) Multiple Affinity Removal System Hu6 column and buffer kit on a HP 1090 HPLC system (Agilent, Santa Clara, USA) according to manufacturer’s instructions. We previously verified by mass spectrometric analysis that this method consistently removes the six targeted proteins only^20^. Here we analyzed unfractionated plasma and high and low abundance protein fractions from four participants by sodium dodecyl sulfate polyacrylamide gel electrophoresis (SDS PAGE) (NuPAGE 4–12% gradient Bis-Tris gels, Life Technologies, Carlsbad, USA) to again verify consistent depletion of the six most abundant proteins across samples (Supplementary Fig. S3). All low abundance protein fractions were buffer exchanged and also analyzed by SDS PAGE (NuPAGE 4–12% gradient Bis-Tris gels, Life Technologies, Carlsbad, USA) prior to tryptic digest and labeling with iTRAQ reagents (Supplementary Fig. S4). For a reference to be included in all multiplex runs, 12 μl plasma each from all NC were pooled, then immunodepleted and prepared as the other samples. Samples were stored at −80 °C until further use.

Labelling of tryptic peptides with iTRAQ 8-plex reagents (Sciex, Framingham, USA) was carried out according to manufacturer’s instructions with slight modifications as outlined in Muenchhoff, *et al.*^20^. iTRAQ-labelled peptides were analysed by LC-MSMS on a LC Packings capillary HPLC system (Thermo Scientific Dionex, Waltham, USA) connected to a TripleTOF 5600^+^ hybrid tandem mass spectrometer (ABSciex, Foster City, USA). Further details on the method are provided in the Supplementary Methods.

### Protein identification and quantitation

LC-MSMS data were processed with Protein Pilot v4.0 (ABSciex, Foster City, USA), applying bias and background correction, and searched against the Swiss-Prot *Homo sapiens* complete proteome to which a list of common contaminant proteins (provided with the Protein Pilot v4.0 software) was added. False discovery rate (FDR) analysis was performed by Protein Pilot v4.0. For statistical analyses, the technical duplicates were processed together in Protein Pilot v4.0 and the protein summaries for the six iTRAQ runs were exported into Excel. The summaries were collated, omitting proteins below 1% FDR, with less than two distinct peptides for identification, from non-human origin (e.g. bovine serum albumin and porcine trypsin contaminants), or without quantitation results.

### Enzyme-linked immunosorbent assay for quantitation of heparin cofactor II

HCII was quantified in the low abundance protein fractions derived from plasma samples using a commercially available ELISA kit (catalogue no. ELH-SERPIND1, RayBiotech, Norcross, USA) according to manufacturer’s instructions. For the assay, all low abundance protein fractions were diluted to 0.4 μg/ml. Samples were assayed in duplicate and the average intra-assay coefficient of variation was 2.7%.

### Neuropsychological testing

Cognitive status was measured using the Clinical Dementia Rating (CDR)^33^,^34^. The CDR scale is widely used in dementia research. Scores of 0, 0.5, 1, 2 and 3 denote no cognitive impairment, very mild, mild, moderate and severe dementia, respectively. In DIAN, asymptomatic MC (aMC) are defined by a CDR score of 0, whereas symptomatic MC (sMC) have a CDR score of ≥0.5. Participants also underwent the Mini Mental State Exam (MMSE), a measure of general cognitive function with scores ranging from 0 (severe impairment) to 30 (no impairment). Scores of ≥24 indicate normal cognitive function^35^. For measures of episodic memory, participants were tested using Logical Memory IA – Immediate Recall (LM-IA) and Logical Memory IIA – Delayed Recall (LM-IIA) from the Wechsler Memory Scale – Revised^36^.

### Neuroimaging

Brain imaging was performed according to ADNI protocols^32^. Details on how Pittsburgh compound B (PiB) and fluorodeoxyglucose (FDG) positron emission tomography (PET) imaging were performed are given in the supplementary information of Bateman *et al.*^2^.

### Statistical analyses

Generalized estimating equations (GEE) method was used to analyse the correlated samples within the six families^37^. Each family was designated as a cluster, resulting in six clusters with sizes of 2, 1, 3, 4, 19 and 6 participants each. We used a linear model with age, gender, *APOE* ε4 status, mutation status and EYO as covariates and the continuous dependent variables were z-transformed. The R geepack (version 1.2-0)^38^,^39^ was used to fit the GEE with exchangeable covariance structure and robust sandwich standard error for the parameter estimates with Gaussian and binomial families respectively for continuous and binary dependent variables. For multiple comparison correction, the false discovery adjusted *p*-values (*q*-values) were obtained using the bioconductor R package qvalue (version 1.34.0)^40^ with default parameter and method^41^. Whenever the global test is significant, a pairwise comparison under the GEE general linear model was done by comparing the base line category with other groups.

### Protein network and pathway analysis

The list of 18 proteins that were differentially abundant in NC and MC at asymptomatic and symptomatic stages was submitted to the web-based bioinformatic tool STRING v9.1^42^ for enrichment analysis against the background of the 81 proteins quantified in all iTRAQ experiments. A network analysis of the 18 proteins was performed using the Core Analysis tool of QIAGEN’s Ingenuity Pathway Analysis (IPA, QIAGEN Redwood City, USA, www.qiagen.com/ingenuity).

## Additional Information

**How to cite this article**: Muenchhoff, J. *et al.* Changes in the plasma proteome at asymptomatic and symptomatic stages of autosomal dominant Alzheimer’s disease. *Sci. Rep.* **6**, 29078; doi: 10.1038/srep29078 (2016).

## Supplementary Material

Supplementary Information

Supplementary Table S2

## Figures and Tables

**Table 1:** Participant characteristics.

Characteristic	NC (*n *= 12)	Asymptomatic MC (*n *= 15)	Symptomatic MC (*n *= 8)
Age, years (SD)	35.2 (10.7)	38.8 (9.4)^a^	44.0 (10.8)
Parental age at onset, years (SD)	50.3 (4.1)	47.3 (7.3)	46.6 (15.1)
Participant’s estimated years to onset, years (SD)	–	−8.5 (7.1)^a^	−2.6 (6.6)^a^
Female, *n* (%)	9 (75.0)	11 (73.3)	5 (62.5)
*APOE* ε4^+^, *n* (%)	2 (16.7)	4 (26.7)	3 (37.5)^a^
Family mutations, *n*			
*PSEN1*	2	6	8
*APPE693Q (Dutch mutation)*	10	9	–
Clinical Dementia Rating score, *n* (%)			
0	11 (91.7)	15 (100)	–
0.5	1 (8.3)	–	5 (62.5)
1	–	–	2 (25.0)
2	–	–	1 (12.5)
3	–	–	–
Mini Mental State Exam score (SD)	27.8 (2.4)	28.0 (1.4)	20.6 (6.2)^a^
Episodic memory			
Immediate recall (LM-IA score)	14.5 (5.4)	16.1 (4.1)	5.0 (6.0)^a^
Delayed recall (LM-IIA score)	13.4 (5.8)	13.9 (5.4)	3.6 (5.7)^a^
Average precuneus thickness, mm (SD)	2.4 (0.1)	2.4 (0.2)	2.0 (0.2)^a^
Glucose metabolism, FDG PET SUVR (SD)^c^			
Precuneus	2.6 (0.2)	2.6 (0.2)^a^	2.0 (0.2)^a^
Caudate nucleus	1.9 (0.1)	1.9 (0.2)^a^	1.8 (0.1)^a^
Aβ deposition, PiB PET SUVR (SD)^c^			
Precuneus	0.6 (0.04)	0.9 (0.4)^a^	2.0 (0.8)^a^
Caudate nucleus	0.5 (0.1)	0.8 (0.5)^a^	1.7 (0.8)^a^
PiB positive, *n* (%)	0 (0)	6 (50.0)	6 (100.0)

*APOE* ε4^+^, at least one apolipoprotein E ε4 allele present; FDG PET SUVR, fluorodeoxyglucose (^18^F) positron emission tomography standard uptake value ratio; PiB PET SUVR, Pittsburgh compound B positron emission tomography standard uptake value ratio; PiB positive, PiB PET SUVR in the precuneus and/or caudate nucleus is ≥0.75.

^a^*p* < 0.05 compared with NC.

^b^Normalised to brainstem.

**Table 2:** Proteins that differed significantly in abundance between NC, aMC and sMC groups.

Biological process	Protein (UniProt accession, gene symbol)	Protein abbreviation	β coefficient	Standard error	*q*-value
Complement system	Complement component C6 (P13671, C6)	C6	−0.35	0.07	1.31 × 10^−5^
Complement system	Complement component C5 (P01031, C5)	C5	0.46	0.11	3.24 × 10^−5^
Complement system	Complement component C3 (P01024, C3)	C3	0.52	0.18	0.0160
Complement system	C4b-binding protein α chain (P04003, C4BPA)	C4BPA	0.38	0.14	0.0272
Inflammatory response	Attractin (O75882, ATRN)	ATRN	−0.84	0.20	3.24 × 10^−4^
Acute phase response	α-1-antichymotrypsin (P01011, SERPINA3)	ACT	−0.32	0.12	0.0341
Acute phase response, mineral balance	α-2-HS-glycoprotein (P02765, AHSG)	AHSG	0.27	0.09	0.0093
Hyaluronan metabolism, inflammation	Inter-α-trypsin inhibitor heavy chain H2 (P19823, ITIH2)	ITIH2	0.38	0.16	0.0490
Hemostasis, acute phase	Histidine-rich glycoprotein (P04196, HRG)	HRG	−0.85	0.20	3.24 × 10^−4^
Hemostasis	Heparin cofactor II (P05546, SERPIND1)	HCII	0.41	0.13	0.0070
Lipid metabolism	Apolipoprotein C-1 (P02654, APOC1)	ApoC1	−0.30	0.07	3.63 × 10^−4^
Lipid metabolism	Apolipoprotein A-I (P02647, APOA1)	ApoA1	−0.29	0.09	0.0044
Lipid metabolism	Apolipoprotein A-IV (P06727, APOA4)	ApoA4	−0.36	0.11	0.0071
Lipid metabolism	Apolipoprotein M (O95445, APOM)	ApoM	−0.46	0.19	0.0414
Protease inhibitor	Protein α-1-microglobulin/bikunin precursor (P02760, AMBP)	AMBP	0.27	0.08	0.0044
Collagen fibril organisation	Lumican (P51884, LUM)	LUM	0.37	0.12	0.0107
Iron homeostasis	Ceruloplasmin (P00450, CP)	CP	−0.23	0.09	0.0386
Thyroid hormone transport	Thyroxine-binding globulin (P05543, SERPINA7)	TBG	0.74	0.30	0.0386

A linear model including age, gender, *APOE* ε4 status, estimated years from expected symptom onset (EYO), mutation and diagnostic status (i.e. NC, aMC and sMC groupings) as covariates was used. Proteins with a *q* value of <0.05 are considered significant. The abundance ratios for these proteins are given in Fig. 1.

## References

[b1] Wimo A. & Prince M. World Alzheimer Report 2010: The Global Economic Impact of Dementia. (Alzheimer’s Disease International, 2010).

[b2] Bateman R. J. *et al.* Clinical and biomarker changes in dominantly inherited Alzheimer’s disease. N Engl J Med 367, 795–804 (2012). 22784036 10.1056/NEJMoa1202753 PMC3474597

[b3] Ryman D. C. *et al.* Symptom onset in autosomal dominant Alzheimer disease: a systematic review and meta-analysis. Neurology 83, 253–260 (2014). 24928124 10.1212/WNL.0000000000000596 PMC4117367

[b4] Reiman E. M. *et al.* Brain imaging and fluid biomarker analysis in young adults at genetic risk for autosomal dominant Alzheimer’s disease in the presenilin 1 E280A kindred: a case-control study. Lancet Neurol 11, 1048–1056 (2012). 23137948 10.1016/S1474-4422(12)70228-4 PMC4181671

[b5] Thomas J. B. *et al.* Functional connectivity in autosomal dominant and late-onset Alzheimer disease. JAMA Neurol 71, 1111–1122 (2014). 25069482 10.1001/jamaneurol.2014.1654 PMC4240274

[b6] Crehan H., Hardy J. & Pocock J. Microglia, Alzheimer’s disease, and complement. Int J Alzheimers Dis 2012, 983640 (2012). 22957298 10.1155/2012/983640 PMC3432348

[b7] Mapstone M. *et al.* Plasma phospholipids identify antecedent memory impairment in older adults. Nat Med 20, 415–418 (2014). 24608097 10.1038/nm.3466 PMC5360460

[b8] Kiddle S. J. *et al.* Candidate blood proteome markers of Alzheimer’s disease onset and progression: a systematic review and replication study. J Alzheimers Dis 38, 515–531 (2014). 24121966 10.3233/JAD-130380

[b9] Sattlecker M. *et al.* Alzheimer’s disease biomarker discovery using SOMAscan multiplexed protein technology. Alzheimers Dement 10, 724–734 (2014). 24768341 10.1016/j.jalz.2013.09.016

[b10] Fortea J. *et al.* Cerebrospinal fluid biomarkers in Alzheimer’s disease families with PSEN1 mutations. Neurodegener Dis 8, 202–207 (2011). 21212633 10.1159/000322229

[b11] Ringman J. M. *et al.* Proteomic changes in cerebrospinal fluid of presymptomatic and affected persons carrying familial Alzheimer disease mutations. Arch Neurol 69, 96–104 (2012). 22232349 10.1001/archneurol.2011.642 PMC3632731

[b12] Wallon D. *et al.* The French series of autosomal dominant early onset Alzheimer’s disease cases: mutation spectrum and cerebrospinal fluid biomarkers. J Alzheimers Dis 30, 847–856 (2012). 22475797 10.3233/JAD-2012-120172

[b13] Fagan A. M. *et al.* Longitudinal change in CSF biomarkers in autosomal-dominant Alzheimer’s disease. Sci Transl Med 6, 226–230 (2014). 10.1126/scitranslmed.3007901 PMC4038930 24598588

[b14] Bateman R. J. *et al.* Autosomal-dominant Alzheimer’s disease: a review and proposal for the prevention of Alzheimer’s disease. Alzheimers Res Ther 3, 1–13 (2011). 21211070 10.1186/alzrt59 PMC3109410

[b15] Kamp J. A. *et al.* Amyloid beta in hereditary cerebral hemorrhage with amyloidosis-Dutch type. Rev Neurosci 25, 641–651 (2014). 24870607 10.1515/revneuro-2014-0008

[b16] Rau J. C., Beaulieu L. M., Huntington J. A. & Church F. C. Serpins in thrombosis, hemostasis and fibrinolysis. J Thromb Haemost 5 Suppl 1, 102–115 (2007). 17635716 10.1111/j.1538-7836.2007.02516.x PMC2670448

[b17] Tolosano E., Fagoonee S., Morello N., Vinchi F. & Fiorito V. Heme scavenging and the other facets of hemopexin. Antioxid Redox Signal 12, 305–320 (2010). 19650691 10.1089/ars.2009.2787

[b18] Hu C. A., Klopfer E. I. & Ray P. E. Human apolipoprotein L1 (ApoL1) in cancer and chronic kidney disease. FEBS Lett 586, 947–955 (2012). 22569246 10.1016/j.febslet.2012.03.002 PMC3349435

[b19] McDade E. *et al.* Cerebral perfusion alterations and cerebral amyloid in autosomal dominant Alzheimer disease. Neurology 83, 710–717 (2014). 25031286 10.1212/WNL.0000000000000721 PMC4150128

[b20] Muenchhoff J. *et al.* Plasma protein profiling of mild cognitive impairment and Alzheimer’s disease across two independent cohorts. J Alzheimers Dis 43, 1355–1373 (2015). 25159666 10.3233/JAD-141266

[b21] Song F. *et al.* Plasma protein profiling of Mild Cognitive Impairment and Alzheimer’s disease using iTRAQ quantitative proteomics. Proteome Sci 12, 5 (2014). 24433274 10.1186/1477-5956-12-5 PMC3898732

[b22] Cutler P. *et al.* Proteomic identification and early validation of complement 1 inhibitor and pigment epithelium-derived factor: Two novel biomarkers of Alzheimer’s disease in human plasma. Proteomics Clin Appl 2, 467–477 (2008). 21136851 10.1002/prca.200780101

[b23] Rembach A. *et al.* Longitudinal Analysis of Serum Copper and Ceruloplasmin in Alzheimer’s Disease. J Alzheimers Dis 34, 171–182 (2013). 23168449 10.3233/JAD-121474

[b24] O’Bryant S. E. *et al.* Biomarkers of Alzheimer’s disease among Mexican Americans. J Alzheimers Dis 34, 841–849 (2013). 23313927 10.3233/JAD-122074 PMC3608404

[b25] Nazeri A. *et al.* Imaging proteomics for diagnosis, monitoring and prediction of Alzheimer’s disease. Neuroimage 102 Pt 2, 657–665 (2014). 25173418 10.1016/j.neuroimage.2014.08.041 PMC6581536

[b26] Whatley B. R., Li L. & Chin L. S. The ubiquitin-proteasome system in spongiform degenerative disorders. Biochim Biophys Acta 1782, 700–712 (2008). 18790052 10.1016/j.bbadis.2008.08.006 PMC2612938

[b27] Huang S. S. *et al.* Plasma heparin cofactor II activity is an independent predictor of future cardiovascular events in patients after acute myocardial infarction. Coron Artery Dis 19, 597–602 (2008). 18971786 10.1097/MCA.0b013e3283155579

[b28] Ikeda Y. *et al.* Heparin cofactor II, a serine protease inhibitor, promotes angiogenesis via activation of the AMP-activated protein kinase-endothelial nitric-oxide synthase signaling pathway. J Biol Chem 287, 34256–34263 (2012). 22904320 10.1074/jbc.M112.353532 PMC3464533

[b29] Aihara K., Azuma H., Akaike M., Sata M. & Matsumoto T. Heparin cofactor II as a novel vascular protective factor against atherosclerosis. J Atheroscler Thromb 16, 523–531 (2009). 19729870 10.5551/jat.1552

[b30] Gupta V. B. *et al.* Plasma apolipoprotein E and Alzheimer disease risk: the AIBL study of aging. Neurology 76, 1091–1098 (2011). 21422459 10.1212/WNL.0b013e318211c352

[b31] Morris J. C. *et al.* Developing an international network for Alzheimer research: The Dominantly Inherited Alzheimer Network. Clin Investig 2, 975–984 (2012). 10.4155/cli.12.93 PMC3489185 23139856

[b32] Mueller S. G. *et al.* The Alzheimer’s disease neuroimaging initiative. Neuroimaging Clin N Am 15, 869–877 (2005). 16443497 10.1016/j.nic.2005.09.008 PMC2376747

[b33] Mckhann G. *et al.* Clinical-Diagnosis of Alzheimers-Disease - Report of the Nincds-Adrda Work Group under the Auspices of Department-of-Health-and-Human-Services Task-Force on Alzheimers-Disease. Neurology 34, 939–944 (1984). 6610841 10.1212/wnl.34.7.939

[b34] Morris J. C. The Clinical Dementia Rating (CDR): current version and scoring rules. Neurology 43, 2412–2414 (1993). 8232972 10.1212/wnl.43.11.2412-a

[b35] Crum R. M., Anthony J. C., Bassett S. S. & Folstein M. F. Population-based norms for the Mini-Mental State Examination by age and educational level. JAMA 269, 2386–2391 (1993). 8479064

[b36] Wechsler D. Manual: Wechsler Memory Scale - Revised. (Psychological Corporation, 1987).

[b37] Burton P., Gurrin L. & Sly P. Extending the simple linear regression model to account for correlated responses: An introduction to generalized estimating equations and multi-level mixed modelling. Stat Med 17, 1261–1291 (1998). 9670414 10.1002/(sici)1097-0258(19980615)17:11<1261::aid-sim846>3.0.co;2-z

[b38] Halekoh U., Hojsgaard S. & Yan J. The R Package geepack for Generalized Estimating Equations. J Stat Softw 15, 1–11 (2006).

[b39] Yan J. & Fine J. Estimating equations for association structures. Stat Med 23, 859–874 (2004). 15027075 10.1002/sim.1650

[b40] Dabney A. & Storey J. D. qvalue: Q-value estimation for false discovery rate control. R package version 1.34.0.

[b41] Storey J. D. & Tibshirani R. Statistical significance for genome-wide experiments. Proc Natl Acad Sci USA 100, 9440–9445 (2003). 12883005 10.1073/pnas.1530509100 PMC170937

[b42] Franceschini A. *et al.* STRING v9.1: protein-protein interaction networks, with increased coverage and integration. Nucleic Acids Res 41, D808–815 (2013). 23203871 10.1093/nar/gks1094 PMC3531103

