# Supplementary material for: Changes in the plasma proteome at asymptomatic and symptomatic stages of autosomal dominant Alzheimer’s disease
Source: Sci Rep. 2016 Jul 6;6:29078. doi: 10.1038/srep29078 (PMC4933916; doi:10.1038/srep29078)
Supplement: Supplementary Information [file srep29078-s1.pdf]

Supplementary Information

Changes in the plasma proteome at asymptomatic and symptomatic stages of autosomal dominant Alzheimer's disease

Julia Muenchhoff[1], Anne Poljak[1,2,3], Anbupalam Thalamuthu[1], Veer B. Gupta[4,5], Pratishtha Chatterjee[4,5,6], Mark Raftery[2], Colin L. Masters[7], John C. Morris[8,9,10], Randall J. Bateman[8,9], Anne M. Fagan[8,9], Ralph N. Martins[4,5,6], Perminder S. Sachdev[1,11,*]

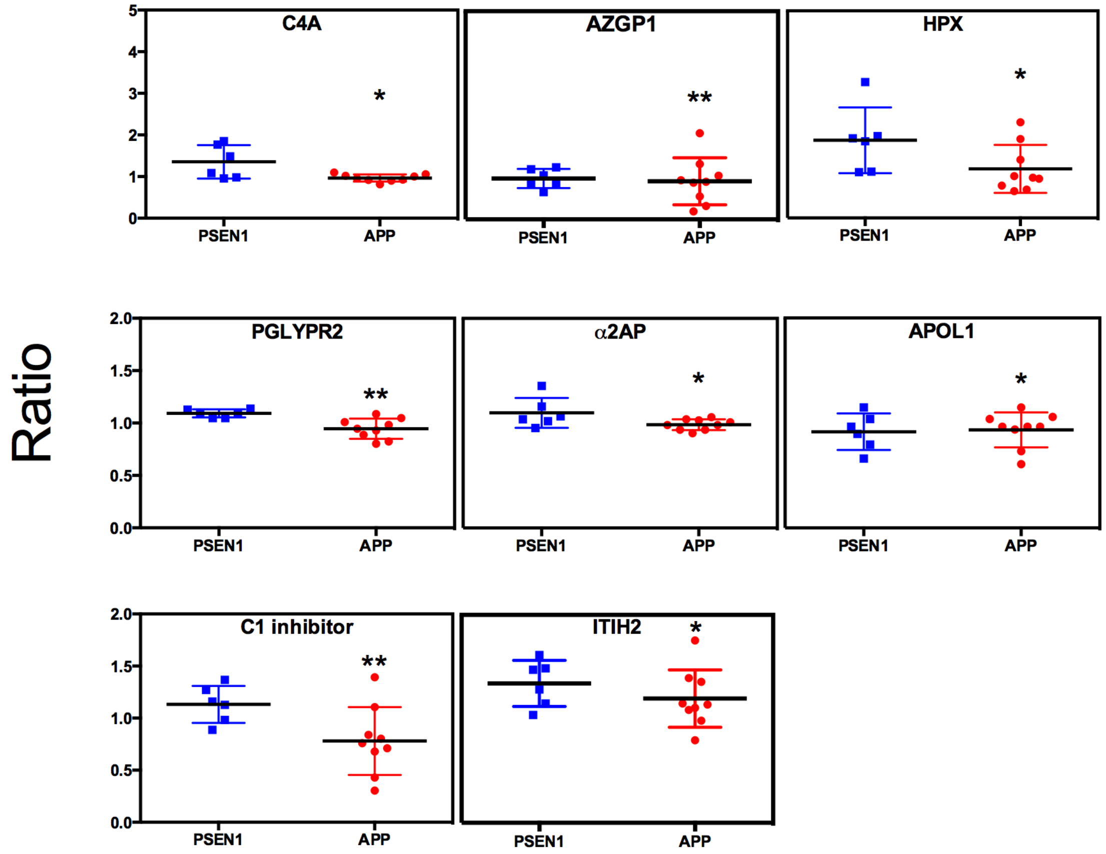

Supplementary Figure S1. Ratios of proteins differentially abundant in asymptomatic carriers of *PSEN1* and *APP* Dutch mutations. Mean ratios and standard deviations of plasma proteins from asymptomatic *PSEN1* mutation carriers (*PSEN1*) and *APP* Dutch mutation carriers (APP) relative to reference masterpool as quantified by iTRAQ. Ratios that significantly differed are marked with asterisks (* $p < 0.05$; ** $p < 0.01$). C4A, complement C4-A; AZGP1, zinc-α-2-glycoprotein; HPX, hemopexin; PGLYPR2, N-acetylmuramoyl-L-alanine amidase isoform 2; α2AP, α-2-antiplasmin; APOL1, apolipoprotein L1; C1 inhibitor, plasma protease C1 inhibitor; ITIH2, inter-α-trypsin inhibitor heavy chain H2.

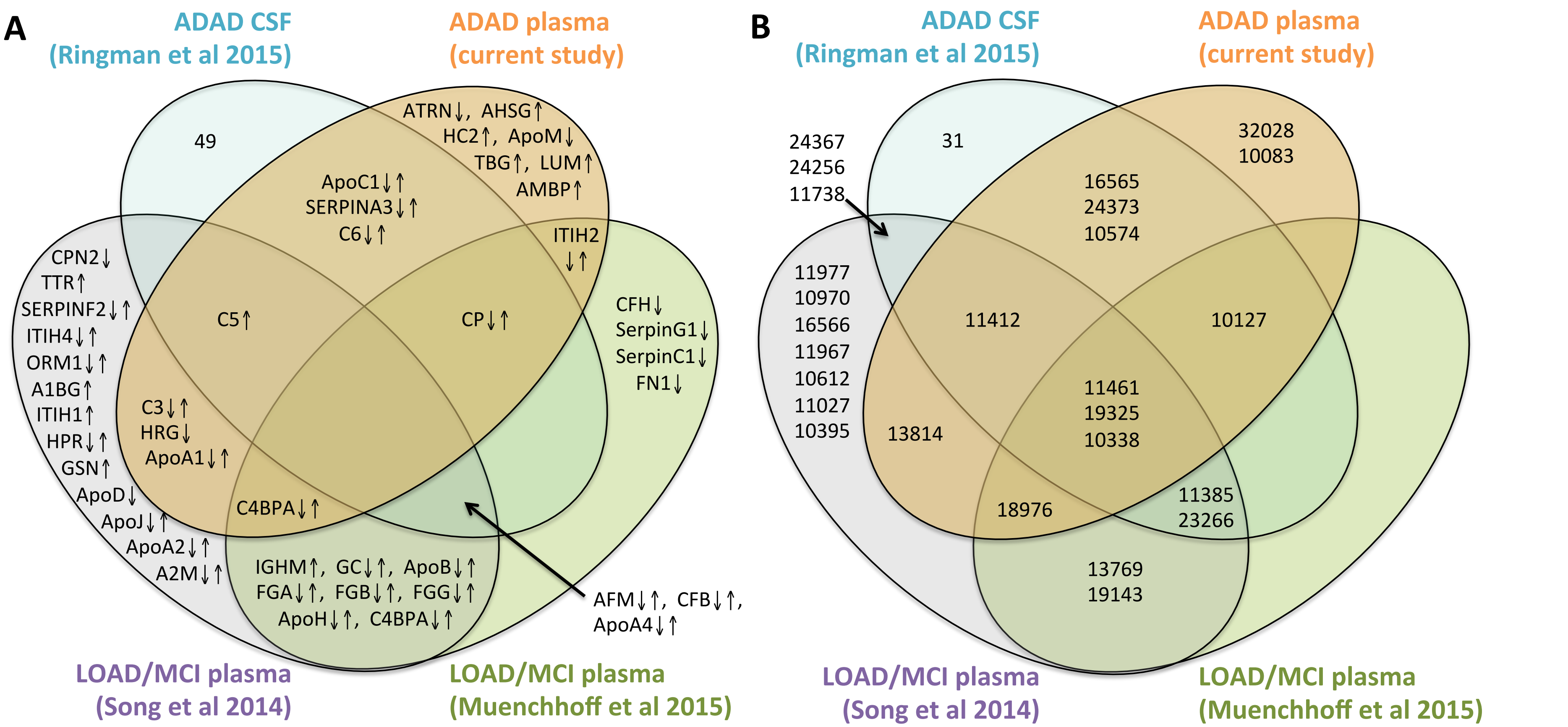

Supplementary Figure S2. Venn diagrams visualising the overlap of proteomic changes between four studies. The diagrams visualise the overlap of individual proteins (**A**) and protein families (**B**) differentially abundant in plasma from ADAD mutation carriers (this study), in CSF from ADAD mutation carriers (Ringman *et al.,* 2012, *Arch Neurol* **69**, 96-104) and in plasma from MCI and LOAD subjects (Song *et al.*, 2012, *Proteome Sci* **12**, 5; Muenchhoff *et al.,* 2015, *J Alzheimers Dis* **43**, 1355-1373). **A**) Arrows indicate increased or decreased levels. For abbreviations see Table 2. **B**) Accession numbers for the PANTHER protein family database are displayed without the PTHR prefix. Full accession numbers and protein family names are: PTHR10083, kunitz-type protease inhibitor-related; PTHR10127, discoidin, cub, egf, laminin , and zinc metalloprotease domain containing; PTHR10338, von willebrand factor, type a domain containing; PTHR10395, uricase and transthyretin-related; PTHR10574, netrin/laminin-related; PTHR10612, apolipoprotein D; PTHR10970, clusterin; PTHR11027, apolipoprotein A-II;PTHR11385, serum albumin-related; PTHR11412, macroglobulin / complement; PTHR11461, serine protease inhibitor, serpin; PTHR11738, MHC class I NK cell receptor; PTHR11967, α-1-acid glycoprotein; PTHR11977, villin; PTHR13769, apolipoprotein B; PTHR13814, fetuin; PTHR16565, apolipoprotein C-I; PTHR16566, apolipoprotein C-II; PTHR18976, apolipoprotein; PTHR19143, fibrinogen/tenascin/angiopoeitin; PTHR19325, complement component-related sushi domain-containing; PTHR23266, immunoglobulin heavy chain; PTHR24256, transmembrane protease, serine; PTHR24367, leucine-rich repeat-containing protein; PTHR24373, PTHR32028, family not named.

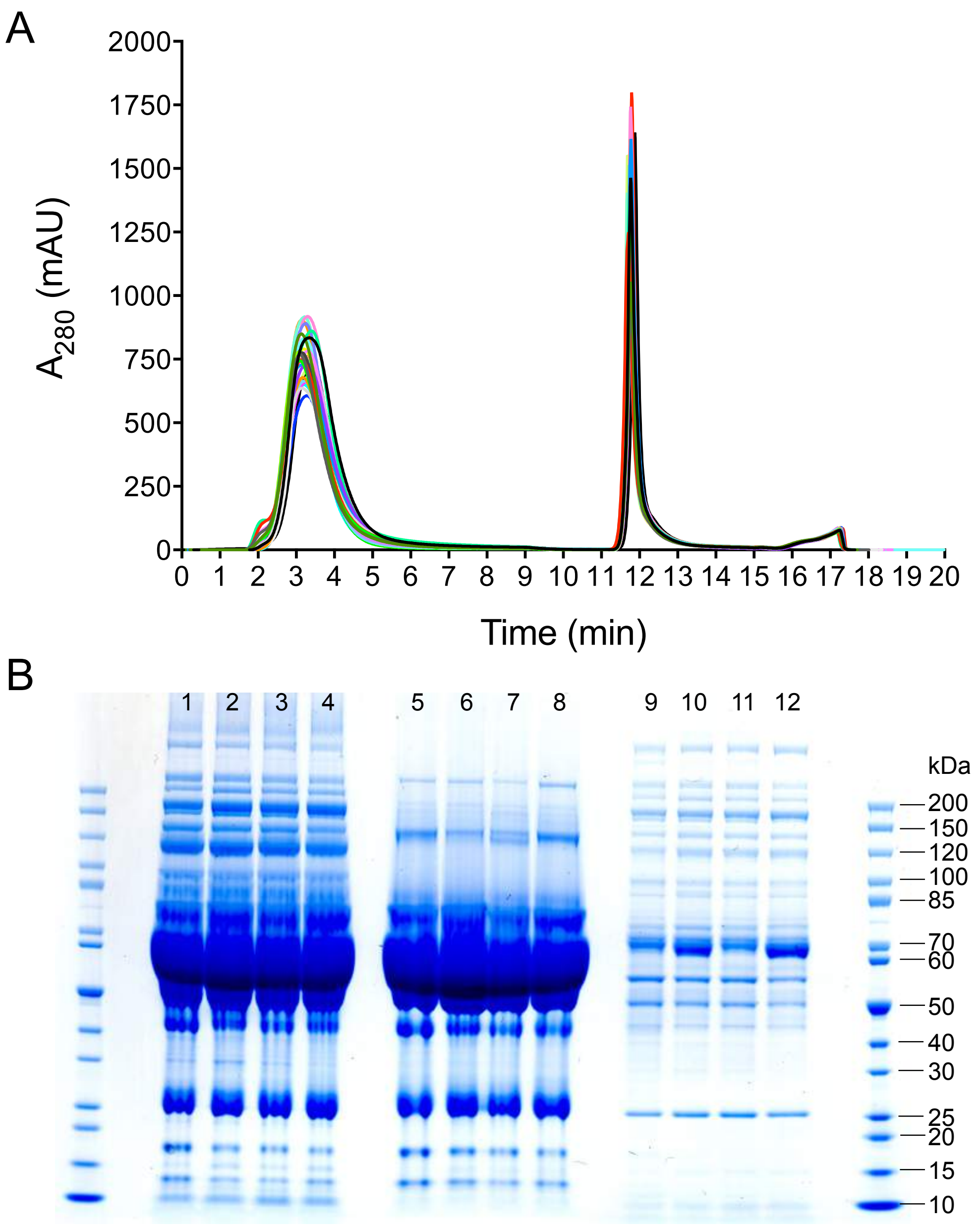

Supplementary Figure S3. Immunodepletion of plasma samples. A) Chromatograms for fractionation of plasma (20 µl) from 35 participants into low and high abundance proteins using the Multiple Affinity Removal System Hu6 column and buffer kit by Agilent (Santa Clara, USA) on a HP 1090 HPLC system (Agilent, Santa Clara, USA). **B)** Colloidal coomassie-stained sodium dodecyl sulfate polyacrylamide gel electrophoresis (NuPAGE 4-12% Bis-Tris, Life Technologies, Carlsbad, CA, USA) of unfractionated plasma proteins (50 µg, lanes 1-4), high abundance plasma proteins (45 µg, lanes 4-8) and low abundance plasma proteins (5 µg, lanes 9-12) from four DIAN participants (one non-carrier, two asymptomatic mutation carriers and one symptomatic mutation carrier who were randomly chosen from each group). The Hu6 column removes the six most abundant proteins equivalent to 85-90% of total plasma protein. Hence, proteins were loaded equivalent to 100% for unfractionated plasma proteins, 90% for the high abundance protein fraction and 10% for the low abundance proteins fraction to better illustrate proportions relative to unfractionated plasma..

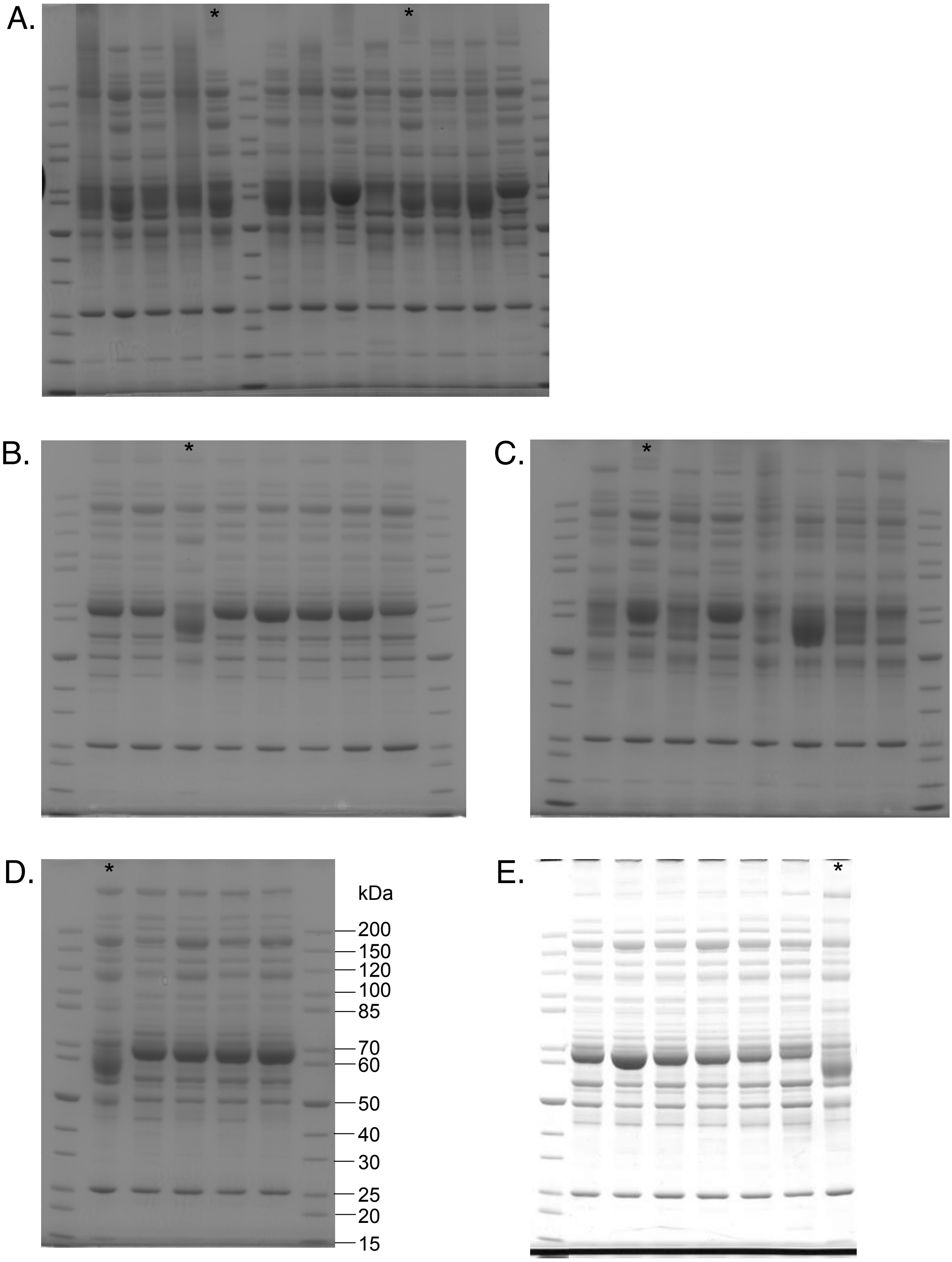

Supplementary Figure S4. Colloidal coomassie-stained sodium dodecyl sulfate polyacrylamide gel electrophoresis (SDS PAGE). SDS PAGE (NuPAGE 4-12% Bis-Tris, Life Technologies, Carlsbad, CA, USA) of low abundance plasma proteins (10 µg) of 35 DIAN participants and non-carrier (NC) masterpool. Samples for iTRAQ multiplex runs 1 and 2 (**A**), 3 (**B**), 4 (**C**)**,** 5 (**D**) and 6 (**E**). The lane corresponding to the NC masterpool in each iTRAQ is labeled with an asterisk. The different format of the SDS PAGE gel in **E** is due to the use of a different gel imaging system.

Supplementary Table S1. Protein summary results for ProteinPilot v4.0 analysis of all iTRAQ multiplex experiments. Numbers for proteins detected refer to all proteins identified at 1% FDR or 95% confidence. Hence, this includes contaminating proteins (e.g. porcine trypsin), proteins with no quantification data or only one distinct peptide for identification. In contrast, Supplementary Table S2 lists proteins used in statistical analyses, which all have quantification data and a minimum of two distinct peptides for identification.

iTRAQ	Proteins detected[a]	Proteins detected[b]	Proteins before grouping[b]	Distinct peptides[b]	Spectra identified[b]	%total spectra
1	105	103	220	5607	18117	23.9
2	109	100	202	5865	21722	29.1
3	115	112	251	6202	23566	31.5
4	97	103	235	5940	21475	28.7
5	102	98	508	5581	20137	26.8
6	125	125	297	6367	22791	30.4

[a]1% FDR
[b]95% confidence, equal to Protein Pilot Unused Score of 1.3.

Supplementary Table S3. Proteins that differed significantly in abundance between asymptomatic carriers of *PSEN1* mutations and noncarriers or *APP* Dutch mutation carriers and noncarriers. A linear model including age, gender, *APOE* ε4 status, estimated years from expected symptom onset (EYO) and mutation type/status (i.e. noncarrier, *PSEN1* and *APP* groupings) as covariates was used (see Materials and Methods section in the main text for details on the statistical analysis). Proteins with a *q* value of < 0.05 are considered significant and marked with an asterisk. Proteins marked in bold also differed significantly in abundance in NC, aMC and sMC group comparisons.

			NC vs PSEN1			NC vs APP		
**Biological process**	**Protein (UniProt accession)**	**Gene symbol**	**β coef-ficient**	**Standard error**	***q*-value**	**β coef-ficient**	**Standard error**	***q*-value**
**Collagen fibril organisation**	**Lumican (P51884)**	**LUM**	**1.52**	**0.31**	**1.22×10^{-5}***	**0.57**	**0.34**	**0.2565**
Lipid metabolism	Apolipoprotein L1 (O14791)	APOL1	-1.23	0.45	0.0216*	0.49	0.38	0.3785
Heme transport	Hemopexin (P02790)	HPX	0.93	0.37	0.0338*	-0.11	0.11	0.4456
**Thyroid hormone transport**	**Thyroxine-binding globulin (P05543)**	**SERPINA7**	**0.61**	**0.25**	**0.0371***	**0.57**	**0.38**	**0.3105**
Vascular function	Plasma kallikrein (P03952)	KLKB1	0.97	0.20	1.22×10^{-5}*	0.64	0.70	0.4456
Blood coagulation, vascular function	Kininogen-1 (P01042)	KNG1	0.75	0.32	0.0371*	0.18	0.35	0.5357
Acute phase, cell chemotaxis	Serum amyloid A-4 protein (P35542)	SAA4	-1.02	0.21	1.22×10^{-5}*	-0.29	0.25	0.4095
Innate immune response	Isoform 2 of N-acetylmuramoyl-L-alanine amidase (Q96PD5-2)	PGLYRP2	0.87	0.21	0.0002*	-0.25	0.32	0.4456
Immune response	Ig γ-3 chain C region (P01860)	IGHG3	0.92	0.38	0.0368*	0.33	0.42	0.4456
Complement system	Plasma protease C1 inhibitor (P05155)	SERPING1	1.57	0.34	3.34×10^{-5}*	0.05	0.26	0.5873
Complement system	Complement C4-B (P0C0L5)	C4B	1.08	0.36	0.0127*	0.00	0.22	0.6228
Complement system	Complement C4-A (P0C0L4)	C4A	1.63	0.58	0.0167*	0.08	0.12	0.4880
Complement system	Complement factor H (P08603)	CFH	0.56	0.24	0.0371*	0.47	0.26	0.2181
**Complement system**	**Complement C3 (P01024)**	**C3**	**1.53**	**0.53**	**0.0162***	**0.83**	**0.30**	**0.0223***
Acute phase, angiogenesis, cell adhesion, cell shape	Fibronectin (P02751)	FN1	1.30	0.44	0.0156*	0.94	0.37	0.0400*
Acute phase, fibrinolysis, platelet activation	α-2-antiplasmin (P08697)	SERPINF2	1.29	0.54	0.0371*	-0.29	0.09	0.0065*
Vascular function	Kallistatin (P29622)	SERPINA4	0.30	0.45	0.2995	-0.25	0.10	0.0439*
**Hemostasis**	**Heparin cofactor 2 (P05546)**	**SERPIND1**	**0.85**	**0.73**	**0.2250**	**0.44**	**0.06**	**9.58×10^{-11}***
Complement system	C4b-binding protein beta chain (P20851)	C4BPB	0.04	0.25	0.3561	0.31	0.09	0.0030*
Complement system	Complement factor I (P05156)	CFI	-0.30	0.61	0.3204	0.55	0.16	0.0041*
Complement system	Complement C1r subcomponent (P00736)	C1R	-0.14	0.35	0.3204	-0.60	0.21	0.0223*
**Complement system**	**Complement component C6 (P13671)**	**C6**	**-0.35**	**0.38**	**0.2421**	**-0.65**	**0.09**	**5.73×10^{-12}***

			NC vs PSEN1			NC vs APP		
**Biological process**	**Protein (UniProt accession)**	**Gene symbol**	**β coef-ficient**	**Standard error**	**q-value**	**β coef-ficient**	**Standard error**	**q-value**
Immune response	Zinc-α-2-glycoprotein (P25311)	AZGP1	0.27	0.19	0.2003	-0.76	0.10	2.91×10^{-12}*
**Inflammatory response**	**Attractin (O75882)**	**ATRN**	**-0.74**	**0.53**	**0.2003**	**-0.98**	**0.22**	**6.25×10^{-5}***
Retinol transport	Retinol-binding protein 4 (P02753)	RBP4	-0.17	0.43	0.3204	-0.73	0.06	$<1.00\times10^{-13}$*
Vitamin E transport	Afamin (P43652)	AFM	0.40	0.71	0.3204	0.84	0.09	$<1.00\times10^{-13}$*
Positive regulation of neurogenesis	Pigment epithelium-derived factor (P36955)	SERPINF1	-0.19	0.37	0.3204	-0.94	0.14	1.12×10^{-10}*

Supplementary Table S4. β coefficients, standard errors and p-values for associations of proteins with glucose metabolism in the precuneus (FDG PET precuneus) and/or caudate nucleus (FDG PET caudate nucleus). A linear model including age, gender, *APOE* ε4 status, estimated years from expected symptom onset (EYO) and mutation status as covariates was used (see Materials and Methods section in the main text for details on the statistical analysis).

		FDG PET caudate nucleus			FDG PET precuneus		
**Protein (UniProt accession)**	**Gene symbol**	**β coefficient**	**Standard error**	**p-value**	**β coefficient**	**Standard error**	**p-value**
α-1B-glycoprotein (P04217)	A1BG	2.12	0.34	2.55×10^{-10}*	1.07	0.43	0.0128
Apolipoprotein E (P02649)	APOE	0.06	0.45	0.8948	0.35	0.09	3.78×10^{-5}*
Apolipoprotein M (O95445)	APOM	0.39	0.97	0.6892	1.85	0.26	1.88×10^{-12}*
Attractin (O75882)	ATRN	7.30	1.60	5.19×10^{-6}*	-0.20	1.14	0.8628
Complement component C4-A (P0C0L4)	C4A	1.15	0.34	0.0007	0.99	0.24	4.53×10^{-5}*
Complement component C6 (P13671)	C6	2.13	0.48	8.85×10^{-6}*	0.50	0.50	0.3177
Fibronectin (P02751)	FN1	0.27	0.57	0.6394	0.74	0.17	1.27×10^{-5}*
Hemoglobin subunit β (P68871)	HBB	0.61	0.05	$<1.00\times10^{-14}$*	0.06	0.05	0.2180
Histidine-rich glycoprotein (P04196)	HRG	2.09	0.36	7.25×10^{-9}*	0.56	0.41	0.1778
Ig γ-3 chain C region (P01860)	IGHG3	-1.59	0.36	7.86×10^{-9}*	-0.33	0.24	0.1651
Ig μ chain C region (P01871)	IGHM	-1.69	0.36	2.02×10^{-6}*	-0.29	0.36	0.4276
Kininogen-1 (P01042)	KNG1	-0.71	0.31	0.0233	0.20	0.12	0.1064
Isoform LMW of Kininogen-1 (P01042-2)	KNG1	0.89	0.31	1.98×10^{-6}*	0.20	0.12	0.0245
Lumican (P51884)	LUM	-5.92	1.64	2.98×10^{-4}*	-0.85	0.51	0.0971
Pigment epithelium-derived factor (P36955)	SERPINF1	4.63	1.14	4.98×10^{-5}*	0.66	1.30	0.6129
Plasma kallikrein (P03952)	KLKB1	-2.52	0.32	6.00×10^{-15}*	0.11	0.58	0.8467
Plasma protease C1 inhibitor (P05155)	SERPING1	-0.95	0.56	0.0876	0.67	0.16	2.26×10^{-5}*
Thyroxine-binding globulin (P05543)	SERPINA7	0.81	0.37	0.0268	-1.20	0.34	4.56×10^{-4}*

*Association is significant at Bonferroni corrected $p < 0.05/81$.

Supplementary Table S5. β coefficients, standard errors and p-values for associations of proteins with amyloid deposition in the precuneus (PiB PET precuneus) and/or caudate nucleus (PiB PET caudate nucleus). A linear model including age, gender, *APOE* ε4 status, estimated years from expected symptom onset (EYO) and mutation status as covariates was used (see Materials and Methods section in the main text for details on the statistical analysis).

		PiB PET caudate nucleus			PiB PET precuneus		
**Protein (UniProt accession)**	**Gene symbol**	**β coefficient**	**Standard Error**	**p-value**	**β coefficient**	**Standard Error**	**p-value**
Complement component C4-A (P0C0L4)	C4A	-1.46	0.20	1.48×10^{-13}*	-1.32	0.19	7.84×10^{-12}*
Complement component C8 β chain (P07358)	C8B	0.21	0.06	4.39×10^{-4}*	0.26	0.09	0.0033
Complement factor I (P05156)	CFI	-1.35	0.47	0.0040	-1.28	0.30	1.88×10^{-5}*
Fibrinogen β chain (P02675)	FGB	-0.25	0.07	5.94×10^{-4}*	-0.22	0.11	0.0522
Heparin cofactor 2 (P05546)	SERPIND1	-1.28	0.33	8.98×10^{-5}*	-0.96	0.50	0.0564
Kininogen-1 (P01042)	KNG1	-0.72	0.15	2.12×10^{-6}*	-0.61	0.13	4.83×10^{-16}*
Plasma kallikrein (P03952)	KLKB1	-1.71	0.46	2.18×10^{-4}*	-1.50	0.69	0.0301
Tetranectin (P05452)	CLEC3B	0.82	0.21	1.05×10^{-4}*	0.83	0.19	1.81×10^{-5}*
Thyroxine-binding globulin (P05543)	SERPINA7	0.68	0.33	0.0385	0.83	0.22	1.19×10^{-4}*
Vitamin K-dependent protein S (P07225)	PROS1	-3.48	0.45	1.28×10^{-14}*	-3.67	0.48	2.21×10^{-14}*
Zinc-α-2-glycoprotein (P25311)	AZGP1	0.52	0.18	0.0031	0.61	0.17	4.35×10^{-4}*

*Association is significant at Bonferroni corrected $p < 0.05/81$.

Supplementary Table S6. β coefficients, standard errors and p-values for associations of proteins with average precuneus thickness. A linear model including age, gender, *APOE* ε4 status, estimated years from expected symptom onset (EYO) and mutation status as covariates was used (see Materials and Methods section in the main text for details on the statistical analysis).

		Average precuneus thickness		
**Protein (UniProt accession)**	**Gene symbol**	**β coefficient**	**Standard Error**	**p-value**
α-2-antiplasmin (P08697)	SERPINF2	2.41	0.63	$1.21{\times}10^{-4}$*
Apolipoprotein A-I (P02647)	APOA1	1.48	0.42	$4.24{\times}10^{-4}$*
Complement C1r subcomponent (P00736)	C1R	-1.68	0.30	$1.38{\times}10^{-8}$*
Complement component C4-A (P0C0L4)	C4A	1.07	0.30	$4.06{\times}10^{-4}$*
Complement factor B (P00751)	CFB	-0.68	0.20	$5.05{\times}10^{-4}$*
Fibronectin (P02751)	FN1	0.85	0.16	$1.41{\times}10^{-4}$*

*Association is significant at Bonferroni corrected $p < 0.05/81$.

Supplementary Table S7. β coefficients, standard errors and p-values for associations of proteins with MMSE score and/or episodic memory represented by LM-IA and LM-IIA scores. A linear model including age, gender, *APOE* ε4 status, estimated years from expected symptom onset (EYO) and mutation status as covariates was used (see Materials and Methods section in the main text for details on the statistical analysis).

		MMSE			LM-IA			LM-IIA		
**Protein (UniProt accession)**	**Gene symbol**	**β coefficient**	**Standard Error**	**p-value**	**β coefficient**	**Standard Error**	**p-value**	**β coefficient**	**Standar d Error**	**p-value**
Complement component C4-A (P0C0L4)	C4A	1.08	0.13	$<1.00\times10^{-14}$*	1.30	0.39	0.0008	1.27	0.32	6.15×10^{-5}*
Hemoglobin subunit β (P68871)	HBB	0.61	0.08	1.82×10^{-13}*	0.43	0.18	0.0144	0.68	0.11	1.44×10^{-9}*
Lumican (P51884)	LUM	-2.86	0.54	1.47×10^{-7}*	-1.08	0.74	0.1445	-0.77	0.78	0.3287
Coagulation factor XII (P00748)	F12	0.65	0.15	1.80×10^{-5}*	0.75	0.26	0.0040	0.76	0.25	0.0021
α-2-HS-glycoprotein (P02765)	AHSG	-0.68	0.20	0.0008	-0.93	0.28	0.0010	-0.82	0.21	6.59×10^{-5}*
Vitronectin (P04004)	VTN	-1.03	0.46	0.0245	-1.39	0.54	0.0106	-1.47	0.39	1.91×10^{-4}*
Complement component C2 (P06681)	C2	-1.21	0.21	1.82×10^{-8}*	-0.83	0.92	0.3634	-0.84	0.94	0.3683
C4b-binding protein β chain (P20851)	C4BPB	-0.44	1.25	0.7228	3.54	0.84	2.69×10^{-5}*	3.82	0.70	5.03×10^{-8}*
Kallistatin (P29622)	SERPINA4	-1.18	0.27	1.22×10^{-5}*	-0.93	0.50	0.0642	-0.65	0.45	0.1460

*Association is significant at Bonferroni corrected $p < 0.05/81$.

Supplementary Table S8. Plasma levels of heparin cofactor II (HCII) for non-carriers (NC), asymptomatic (aMC) and symptomatic mutation carriers (sMC). HCII was quantified using ELISA in the low abundance protein fractions derived from plasma and used in iTRAQ experiments. Protein levels are expressed as µg protein of interest per mg total protein in the sample.

	**HCII**
NC (n = 12), µg per mg protein (SD)	16.73 (3.31)
aMC (n = 15), µg per mg protein (SD)	17.35 (3.99)
sMC (n = 2), µg per mg protein (SD)	16.42 (3.47)
Correlation with iTRAQ data, r (p-value)	0.58 (<0.001)

Supplementary Methods

Plasma immunodepletion and sample preparation

The six most abundant plasma proteins (albumin, transferrin, immunoglobulins G and A, haptoglobin and antitrypsin) were immunodepleted from plasma samples (20 µl) using the Agilent (Santa Clara, USA) Multiple Affinity Removal System Hu6 column and buffer kit on a HP 1090 HPLC system (Agilent, Santa Clara, USA) according to manufacturer's instructions. We previously verified that this method only removes the six targeted proteins [1]. The depleted fractions were buffer exchanged and concentrated into 20 mM $NaHCO_3$ using Amicon 3 kDa centrifugal devices (Millipore, Billerica, USA). Total protein was quantified by absorbance measurements (280 nm) with a ND-1000 spectrophotometer (NanoDrop Technologies, Wilmington, USA), using the relationship; A_{280} of 1 = 1 mg mL^{-1}. Low abundance protein fractions were analyzed by sodium dodecyl sulfate polyacrylamide gel electrophoresis (SDS PAGE) (NuPAGE 4-12% gradient Bis-Tris gels, Life Technologies, Carlsbad, USA) to verify consistent depletion of the six most abundant proteins across all samples (Supplementary Fig. S3 and S4). Samples were stored at -80 °C until further use.

iTRAQ-labelling and purification of labelled peptides

Labelling of tryptic peptides with iTRAQ 8-plex reagents (Sciex, Framingham, USA) was carried out according to manufacturer's instructions with slight modifications as outlined in Muenchhoff, *et al.* [1]. Briefly, 50 µg of the low abundance proteins from each plasma sample were reduced with *tris* (2-carboxyethyl) phosphine, alkylated with iodoacetamide and digested with trypsin overnight at 37°C. Tryptic peptides were combined with iTRAQ reagents and the pH-adjusted labelling reaction was allowed to proceed for 2 hrs at room temperature. iTRAQ-labelled peptides were combined and passed through a cation exchange cartridge to remove excess reagents. The eluent was evaporated to dryness, resuspended in 500 µl 0.2% heptafluorobutyric acid (HFBA) and purified using a C18 macrotrap (Michrom Bioresources, Auburn, USA). To ensure maximal recovery of labelled peptides, the C18 flow through was passed through an Oasis cartridge to capture any peptides not bound on the macrotrap. The eluents from both cartridges were combined, evaporated to dryness and resuspended in 100 µl 0.05% HFBA, 1% formic acid for liquid chromatography tandem mass spectrometry (LC-MSMS) analysis.

LC-MSMS

iTRAQ-labelled peptides were analysed by LC-MSMS. LC was carried out on a LC Packings capillary HPLC system, comprised of a Dionex UltiMate 3000 RSLCnano pump system, Switchos valve unit and Famos autosampler (Thermo Scientific Dionex, Waltham, USA). The resuspended iTRAQ labelled peptides were injected onto a C18 precolumn cartridge (Acclaim PepMap 100, 5 µm 100 Å, Thermo Scientific Dionex, Waltham, USA), which was washed for 10 min prior to switching inline to a capillary column (10 cm) containing C18 reverse phase packing material (Reprosil-Pur, 1.9 µ, 200 Å, Dr. Maisch GmbH, Ammerbuch-Entringen, Germany). Peptides were eluted using a 240 min gradient of buffer A ($H_2O:CH_3CN$ of 98:2 containing 0.1% formic acid) to buffer B ($H_2O:CH_3CN$ of 20:80 containing 0.1% formic acid) at 200 nL/min. High voltage (2300 V) was applied through a low volume tee (Upchurch Scientific, Oak Harbor, USA) at the column inlet and the outlet positioned ~1 cm from the orifice of a TripleTOF 5600^+ hybrid tandem mass spectrometer (ABSciex, Foster City, USA). Positive ions were generated by electrospray ionization and the TripleTOF 5600^+ system operated in information-dependent acquisition mode. A time of flight MS survey scan was acquired (*m/z* 375-1600, 0.4 s) and up to ten multiply charged ions (*m/z* 375-1250, counts > 200, charge state $\geq 2^+$ and $\leq 5^+$) sequentially selected by Q1 for MSMS analysis. Nitrogen was used as collision gas and an optimum collision energy automatically chosen (based on charge state and mass). Tandem mass spectra were accumulated for 0.3 s. LC-MSMS was performed twice for analytical replicates.

Supplementary Discussion

Proteins in inflammation

Four components of the complement system were found differentially abundant in NC, aMC and sMC, namely, C3, C5, C6 and C4b-binding protein α chain. There is evidence to indicate that the complement system might play a crucial role in AD pathology. Aβ interacts with components of the classical and alternate pathway, activating both in the AD brain [2,3]. Activation of the complement system in AD affords some protection in the form of clearance of Aβ, but also causes harm through chronic inflammation leading to tissue damage and lysis of neurons [3]. Many studies have previously suggested complement components as biomarkers for AD (e.g. [1,4-7]).

ACT, one of three serine protease inhibitors (SERPIN) found differentially abundant, is an acute phase protein, regulating protease activity of neutrophil cathepsin G, mast cell chymase and others during inflammation, thereby preventing tissue damage [8]. It might also be involved in atherosclerotic processes and stabilisation of the aorta as indicated by its differential vascular expression in atherosclerotic lesions and abdominal aortic aneurysms [9]. Its expression is elevated in the AD brain, it co-localises with amyloid plaques and might induce tau hyperphosphorylation [10,11]. *In vivo* studies in transgenic mouse models report accelerated amyloid plaque formation upon expression of human ACT [10,12-16], which could potentially be due to ACT inhibiting a serine protease involved in Aβ degradation [17]. Although controversial, variation in the SERPINA3 promoter and ACT protein sequence have also been associated with increased risk of LOAD [18-20]. A number of reports suggested ACT levels in plasma/serum as a biomarker for LOAD (see [21] for a review).

AHSG also known as fetuin-A is a multifunctional negative acute phase protein that can bind cations, such as calcium. It is involved in regulation of mineralization and ostoegenesis, insulin signaling as well as inflammation [22]. AHSG serum/plasma levels are known to predict incident type 2 diabetes and vascular disease risk due to its inhibitory action on vascular calcification [23]. In a rodent model, peripherally administered bovine AHSG protected against early cerebral ischemic injury, likely due to its anti-inflammatory properties [24]. Due to its links to vascular disease and neuroinflammation, both components of AD pathology, AHSG has been proposed as a biomarker for LOAD in plasma and CSF, and was shown to associate with severity of cognitive decline [23,25-27]. Individuals homozygous for the AHSG 1 allele in an Italian population were found to be at nearly four times greater risk of developing LOAD [28].

Protein α-1-microglobulin/bikunin precursor is the precursor for the structurally and functionally unrelated proteins α-1-microglobulin and bikunin. α-1-microglobulin is a member of the lipocalin superfamily, a group of proteins that carry lipophilic ligands. It may protect cells from oxidative stress by transporting heme groups and free radicals released from hemoglobin from cytosols and extravascular fluids to the kidneys [29]. It also negatively regulates the immune response of lymphocytes, T-cells and granulocytes [30-32]. α-1-microglobulin levels were found to differ in the plasma from AD and control patients [33] and associated with brain atrophy in AD patients [34]. Bikunin, also known as inter-α-trypsin inhibitor light chain, is a Kunitz-type protease inhibitor possibly involved in endothelial cell growth and extracellular matrix stabilisation [35,36]. Proteins in the inter-α-trypsin inhibitor family consist of the common light chain protein bikunin linked to one or two of various heavy chains (H1-4) by a chondroitin sulphate chain. Interestingly, significant differences in ITIH2 were also observed here. The protease inhibitory activity of bikunin might prevent inflammation-related proteolytic activity. The inter-α-trypsin inhibitor heavy chains can be transferred to hyaluronan (a major component of the pericellular matrix), resulting in formation of a heavy chain-hyaluronan complex and the release of bikunin, which is then excreted into urine. Heavy chains also interact with components of the complement system to lower levels of the powerful mediator C5a [37]. Changes in abundance of plasma inter-α-trypsin inhibitor heavy chain 2 were previously reported in MCI [1] and LOAD [21,38].

ATRN is the only protein found differentially abundant in the early asymptomatic stage of ADAD but not in the later stage. ATRN is expressed as three isoforms, with isoform 1 having a C-terminal extension that anchors the protein in the membrane, whereas isoforms 2 and 3 are secreted. No peptides specific to any of the three isoforms were detected; hence, no conclusions can be drawn on presence or quantity of the individual isoforms. Expression of the secreted isoforms is down-regulated in the human brain and these isoforms have been shown to disrupt neurite formation *in vitro* [39]. By contrast, the membrane-bound isoform is expressed in the CNS, where it is critical for myelination [39,40]. Rats with loss of function mutations in glycosylated transmembrane ATRN have age-dependent spongiform degeneration, hypomyelination and abnormal ROS metabolism in the brain [41-43]. ATRN is also involved in the regulation of skin pigmentation, energy control and immunity [44,45]. In the immune system, ATRN regulatory activity allows immune cells to interact to form regulatory clusters. This regulatory activity of ATRN might be affected by the balance of membrane-bound and soluble isoforms [46]. The mechanism of ATRN function is not well understood with suggestions of DP4 protease activity for ATRN being disputed [47].

Proteins in hemostasis and vascular health

Two proteins with functions in hemostasis and vascular health were found differentially abundant in the NC, aMC and sMC groups. Both proteins are also able to modulate the immune response, reflecting the close connection of these systems.

HRG modulates a variety of biological processes, including blood coagulation, fibrinolysis, angiogenesis, complement activation and aggregation of immune complexes [48,49]. It exerts its influence via binding of various ligands, e.g. heparin, heparin sulphate, plasminogen and plasmin, fibrinogen, complement component C1q, IgG, Fc γ receptor and divalent cations [50]. Similar to AHSG mentioned above, HRG also belongs to the cystatin type 3 family of proteins, and is able to inhibit the formation of spontaneous apatite calcifications formed from calcium and phosphate ions (both divalent cations) *in vitro*. Hence, it possibly prevents ectopic calcification [50]. Levels of HRG in blood were reported to differ between MCI [5] and AD [51] subjects and healthy controls.

HCII is a SERPIN that in the presence of glucosaminoglycans (e.g. heparin or dermatan sulphate) inhibits thrombin. HCII is particularly relevant in the intima and media of the vascular wall, which is rich in dermatan sulfate [52]. As such, HCII protects against thrombin-induced vascular remodelling and consequently atherosclerosis [53]. It may also promote angiogenesis via an AMP-activated protein kinase-endothelial nitric oxide synthase pathway [54]. Hence, HCII has been suggested as a therapeutic target and potential biomarker for arterial disease [53-55].

Proteins in lipid metabolism

Four apolipoproteins, ApoA1, ApoA4, ApoC1 and ApoM, differed in abundance in NC, aMC and sMC groups. Apolipoproteins are constituents of lipoprotein particles, such as chylomicrons, VLDL, LDL and HDL, which transport lipids between tissues for fuel and cholesterol metabolism. The apolipoproteins serve as carrier, receptor-binding and regulatory proteins in these particles. As such, they are crucial components in lipid metabolism with implications for cardiovascular disease, obesity and diabetes mellitus (for a review see [56]). Recently, the apolipoproteins have also emerged as a protein family of particular interest in AD, since alterations in lipid metabolism have been associated with AD pathology [57,58] and the APOE ε4 allele is the most significant genetic risk factor for LOAD. Furthermore, plasma levels of clusterin (also known as apolipoprotein J) are emerging as a meaningful biomarker for MCI [59] and LOAD [60], and carriers of *CLU* risk alleles show faster rates of cognitive decline [61,62].

References

1 Muenchhoff, J. *et al.* Plasma protein profiling of mild cognitive impairment and Alzheimer's disease across two independent cohorts. *J Alzheimers Dis* **43**, 1355-1373 (2015).

2 Crehan, H., Hardy, J. & Pocock, J. Microglia, Alzheimer's disease, and complement. *Int J Alzheimers Dis* **2012**, 983640 (2012).

3 Veerhuis, R., Nielsen, H. M. & Tenner, A. J. Complement in the brain. *Mol Immunol* **48**, 1592-1603 (2011).

4 Sattlecker, M. *et al.* Alzheimer's disease biomarker discovery using SOMAscan multiplexed protein technology. *Alzheimers Dement* (2014).

5 Song, F. *et al.* Plasma protein profiling of Mild Cognitive Impairment and Alzheimer's disease using iTRAQ quantitative proteomics. *Proteome Sci* **12**, 5 (2014).

6 Aiyaz, M., Lupton, M. K., Proitsi, P., Powell, J. F. & Lovestone, S. Complement activation as a biomarker for Alzheimer's disease. *Immunobiology* **217**, 204-215 (2012).

7 Jayasena, T. *et al.* Upregulation of glycolytic enzymes, mitochondiral dysfunction and increased cytotoxicity in glial cells treated with Alzheimer's disease plasma. *PLoS One* **10**, e0116092 (2015).

8 Baker, C., Belbin, O., Kalsheker, N. & Morgan, K. SERPINA3 (aka alpha-1-antichymotrypsin). *Front Biosci* **12**, 2821-2835 (2007).

9 Wagsater, D. *et al.* Serine protease inhibitor A3 in atherosclerosis and aneurysm disease. *Int J Mol Med* **30**, 288-294 (2012).

10 Tyagi, E., Fiorelli, T., Norden, M. & Padmanabhan, J. Alpha 1-Antichymotrypsin, an Inflammatory Protein Overexpressed in the Brains of Patients with Alzheimer's Disease, Induces Tau Hyperphosphorylation through c-Jun N-Terminal Kinase Activation. *Int J Alzheimers Dis* **2013**, 606083 (2013).

11 Yu, G. & Jia, J. Is there an association of regulatory region polymorphism in the alpha-1-antichymotrypsin gene with sporadic Alzheimer's disease in the northern Han-Chinese population? *J Clin Neurosci* **17**, 766-769 (2010).

12 Nilsson, L. N. *et al.* Alpha-1-antichymotrypsin promotes beta-sheet amyloid plaque deposition in a transgenic mouse model of Alzheimer's disease. *J Neurosci* **21**, 1444-1451 (2001).

13 Mucke, L. *et al.* Astroglial expression of human alpha(1)-antichymotrypsin enhances alzheimer-like pathology in amyloid protein precursor transgenic mice. *Am J Pathol* **157**, 2003-2010 (2000).

14 Ma, J., Brewer, H. B., Jr. & Potter, H. Alzheimer A beta neurotoxicity: promotion by antichymotrypsin, ApoE4; inhibition by A beta-related peptides. *Neurobiol Aging* **17**, 773-780 (1996).

15 Eriksson, S., Janciauskiene, S. & Lannfelt, L. Alpha 1-antichymotrypsin regulates Alzheimer beta-amyloid peptide fibril formation. *Proc Natl Acad Sci U S A* **92**, 2313-2317 (1995).

16 Fraser, P. E., Nguyen, J. T., McLachlan, D. R., Abraham, C. R. & Kirschner, D. A. Alpha 1-antichymotrypsin binding to Alzheimer A beta peptides is sequence specific and induces fibril disaggregation in vitro. *J Neurochem* **61**, 298-305 (1993).

17 Abraham, C. R., McGraw, W. T., Slot, F. & Yamin, R. Alpha 1-antichymotrypsin inhibits A beta degradation in vitro and in vivo. *Ann N Y Acad Sci* **920**, 245-248 (2000).

18 Porcellini, E. *et al.* Haplotype of single nucleotide polymorphisms in exon 6 of the MZF-1 gene and Alzheimer's disease. *J Alzheimers Dis* **34**, 439-447 (2013).

19 Guan, F., Gu, J., Hu, F., Zhu, Y. & Wang, W. Association between alpha1-antichymotrypsin signal peptide -15A/T polymorphism and the risk of Alzheimer's disease: a meta-analysis. *Mol Biol Rep* **39**, 6661-6669 (2012).

20 Dou, C. *et al.* The association of ACT -17 A/T polymorphism with Alzheimer's disease: a meta-analysis. *Curr Alzheimer Res* **10**, 63-71 (2013).

21 Zabel, M. *et al.* Assessing Candidate Serum Biomarkers for Alzheimer's Disease: A Longitudinal Study. *J Alzheimers Dis* **30**, 311-321 (2012).

22 Mukhopadhyay, S., Mondal, S. A., Kumar, M. & Dutta, D. Pro-inflammatory and anti-inflammatory attributes of fetuin-A: a novel hepatokine: modulating cardiovascular and glycemic outcomes in metabolic syndrome. *Endocr Pract* **20**, 1345-1351 (2014).

23 Laughlin, G. A., McEvoy, L. K., Barrett-Connor, E., Daniels, L. B. & Ix, J. H. Fetuin-A, a new vascular biomarker of cognitive decline in older adults. *Clin Endocrinol* **81**, 134-140 (2014).

24 Wang, H. *et al.* Peripheral administration of fetuin-A attenuates early cerebral ischemic injury in rats. *J Cereb Blood Flow Metab* **30**, 493-504 (2010).

25 Smith, E. R., Nilforooshan, R., Weaving, G. & Tabet, N. Plasma fetuin-A is associated with the severity of cognitive impairment in mild-to-moderate Alzheimer's disease. *J Alzheimers Dis* **24**, 327-333 (2011).

26 Wijte, D. *et al.* A novel peptidomics approach to detect markers of Alzheimer's disease in cerebrospinal fluid. *Methods* **56**, 500-507 (2012).

27 Puchades, M. *et al.* Proteomic studies of potential cerebrospinal fluid protein markers for Alzheimer's disease. *Brain Res Mol Brain Res* **118**, 140-146 (2003).

28 Geroldi, D. *et al.* Genetic association of alpha2-Heremans-Schmid glycoprotein polymorphism with late-onset Alzheimer's disease in Italians. *Neurosci Lett* **386**, 176-178 (2005).

29 Olsson, M. G. *et al.* Pathological conditions involving extracellular hemoglobin: molecular mechanisms, clinical significance, and novel therapeutic opportunities for alpha(1)-microglobulin. *Antioxid Redox Signal* **17**, 813-846 (2012).

30 Tyagi, S., Salier, J. P. & Lal, S. K. The liver-specific human alpha(1)-microglobulin/bikunin precursor (AMBP) is capable of self-association. *Arch Biochem Biophys* **399**, 66-72 (2002).

31 Logdberg, L. & Akerstrom, B. Immunosuppressive properties of alpha 1-microglobulin. *Scand J Immunol* **13**, 383-390 (1981).

32 Wester, L., Johansson, M. U. & Akerstrom, B. Physicochemical and biochemical characterization of human alpha 1-microglobulin expressed in baculovirus-infected insect cells. *Protein Expr Purif* **11**, 95-103 (1997).

33 Llano, D. A., Devanarayan, V., Simon, A. J. & Alzheimer's Disease Neuroimaging, I. Evaluation of plasma proteomic data for Alzheimer disease state classification and for the prediction of progression from mild cognitive impairment to Alzheimer disease. *Alzheimer Dis Assoc Disord* **27**, 233-243 (2013).

34 Thambisetty, M. *et al.* Plasma biomarkers of brain atrophy in Alzheimer's disease. *PLoS One* **6**, e28527 (2011).

35 McKeehan, W. L., Sakagami, Y., Hoshi, H. & McKeehan, K. A. Two apparent human endothelial cell growth factors from human hepatoma cells are tumor-associated proteinase inhibitors. *J Biol Chem* **261**, 5378-5383 (1986).

36 Zhuo, L. *et al.* Defect in SHAP-hyaluronan complex causes severe female infertility. A study by inactivation of the bikunin gene in mice. *J Biol Chem* **276**, 7693-7696 (2001).

37 Zhuo, L. & Kimata, K. Structure and function of inter-alpha-trypsin inhibitor heavy chains. *Connect Tissue Res* **49**, 311-320 (2008).

38 Liao, P. C., Yu, L., Kuo, C. C., Lin, C. & Kuo, Y. M. Proteomics analysis of plasma for potential biomarkers in the diagnosis of Alzheimer's disease. *Proteomics Clin Appl* **1**, 506-512 (2007).

39 Tang, W. & Duke-Cohan, J. S. Human secreted attractin disrupts neurite formation in differentiating cortical neural cells in vitro. *J Neuropathol Exp Neurol* **61**, 767-777 (2002).

40 Kuramoto, T. *et al.* Attractin/mahogany/zitter plays a critical role in myelination of the central nervous system. *Proc Natl Acad Sci U S A* **98**, 559-564 (2001).

41 Rehm, S., Mehraein, P., Anzil, A. P. & Deerberg, F. A new rat mutant with defective overhairs and spongy degeneration of the central nervous system: clinical and pathologic studies. *Lab Anim Sci* **32**, 70-73 (1982).

42 Gomi, H., Ueno, I. & Yamanouchi, K. Antioxidant enzymes in the brain of zitter rats: abnormal metabolism of oxygen species and its relevance to pathogenic changes in the brain of zitter rats with genetic spongiform encephalopathy. *Brain Res* **653**, 66-72 (1994).

43 Kondo, A., Sendoh, S., Takamatsu, J. & Nagara, H. The zitter rat: membranous abnormality in the Schwann cells of myelinated nerve fibers. *Brain Res* **613**, 173-179 (1993).
44 Nagle, D. L. *et al.* The mahogany protein is a receptor involved in suppression of obesity. *Nature* **398**, 148-152 (1999).
45 Gunn, T. M. *et al.* The mouse mahogany locus encodes a transmembrane form of human attractin. *Nature* **398**, 152-156 (1999).
46 Duke-Cohan, J. S., Tang, W. & Schlossman, S. F. Attractin: a cub-family protease involved in T cell-monocyte/macrophage interactions. *Adv Exp Med Biol* **477**, 173-185 (2000).
47 Friedrich, D. *et al.* Does human attractin have DP4 activity? *Biol Chem* **388**, 155-162 (2007).
48 Wakabayashi, S. New insights into the functions of histidine-rich glycoprotein. *Int Rev Cell Mol Biol* **304**, 467-493 (2013).
49 Horstman, L. L. *et al.* Complement in neurobiology. *Front Biosci (Landmark Ed)* **16**, 2921-2960 (2011).
50 Jones, A. L., Hulett, M. D. & Parish, C. R. Histidine-rich glycoprotein: A novel adaptor protein in plasma that modulates the immune, vascular and coagulation systems. *Immunol Cell Biol* **83**, 106-118 (2005).
51 Zhang, R. *et al.* Mining biomarkers in human sera using proteomic tools. *Proteomics* **4**, 244-256 (2004).
52 Rau, J. C., Mitchell, J. W., Fortenberry, Y. M. & Church, F. C. Heparin cofactor II: discovery, properties, and role in controlling vascular homeostasis. *Semin Thromb Hemost* **37**, 339-348 (2011).
53 Aihara, K., Azuma, H., Akaike, M., Sata, M. & Matsumoto, T. Heparin cofactor II as a novel vascular protective factor against atherosclerosis. *J Atheroscler Thromb* **16**, 523-531 (2009).
54 Ikeda, Y. *et al.* Heparin cofactor II, a serine protease inhibitor, promotes angiogenesis via activation of the AMP-activated protein kinase-endothelial nitric-oxide synthase signaling pathway. *J Biol Chem* **287**, 34256-34263 (2012).
55 Huang, S. S. *et al.* Plasma heparin cofactor II activity is an independent predictor of future cardiovascular events in patients after acute myocardial infarction. *Coron Artery Dis* **19**, 597-602 (2008).
56 Dominiczak, M. H. & Caslake, M. J. Apolipoproteins: metabolic role and clinical biochemistry applications. *Ann Clin Biochem* **48**, 498-515 (2011).
57 Martins, I. J. *et al.* Cholesterol metabolism and transport in the pathogenesis of Alzheimer's disease. *J Neurochem* **111**, 1275-1308 (2009).
58 Takechi, R., Galloway, S., Pallebage-Gamarallage, M. M., Lam, V. & Mamo, J. C. Dietary fats, cerebrovasculature integrity and Alzheimer's disease risk. *Prog Lipid Res* **49**, 159-170 (2010).
59 Song, F. *et al.* Plasma apolipoprotein levels are associated with cognitive status and decline in a community cohort of older individuals. *PLoS One* **7**, e34078 (2012).
60 Thambisetty, M. *et al.* Association of plasma clusterin concentration with severity, pathology, and progression in Alzheimer disease. *Arch Gen Psychiatry* **67**, 739-748 (2010).
61 Yu, J. T. & Tan, L. The role of clusterin in Alzheimer's disease: pathways, pathogenesis, and therapy. *Mol Neurobiol* **45**, 314-326 (2012).
62 Thambisetty, M. *et al.* Alzheimer risk variant CLU and brain function during aging. *Biol Psychiatry* **73**, 399-405 (2013).
